# Spectrin-based membrane skeleton supports ciliogenesis

**DOI:** 10.1371/journal.pbio.3000369

**Published:** 2019-07-12

**Authors:** Ru Jia, Dongdong Li, Ming Li, Yongping Chai, Yufan Liu, Zhongyun Xie, Wenxin Shao, Chao Xie, Liuju Li, Xiaoshuai Huang, Liangyi Chen, Wei Li, Guangshuo Ou

**Affiliations:** 1 Tsinghua-Peking Center for Life Sciences, Beijing Frontier Research Center for Biological Structure, School of Life Sciences and MOE Key Laboratory for Protein Science, Tsinghua University, Beijing, China; 2 State Key Laboratory of Membrane Biology, Beijing Key Laboratory of Cardiometabolic Molecular Medicine, Institute of Molecular Medicine, Peking University, Beijing, China; 3 School of Medicine, Tsinghua University, Beijing, China; Brandeis University, UNITED STATES

## Abstract

Cilia are remarkable cellular devices that power cell motility and transduce extracellular signals. To assemble a cilium, a cylindrical array of 9 doublet microtubules push out an extension of the plasma membrane. Membrane tension regulates cilium formation; however, molecular pathways that link mechanical stimuli to ciliogenesis are unclear. Using genome editing, we introduced hereditary elliptocytosis (HE)- and spinocerebellar ataxia (SCA)-associated mutations into the *Caenorhabditis elegans* membrane skeletal protein spectrin. We show that these mutations impair mechanical support for the plasma membrane and change cell shape. RNA sequencing (RNA-seq) analyses of spectrin-mutant animals uncovered a global down-regulation of ciliary gene expression, prompting us to investigate whether spectrin participates in ciliogenesis. Spectrin mutations affect intraflagellar transport (IFT), disrupt axonemal microtubules, and inhibit cilium formation, and the endogenous spectrin periodically distributes along cilia. Mammalian spectrin also localizes in cilia and regulates ciliogenesis. These results define a previously unrecognized yet conserved role of spectrin-based mechanical support for cilium biogenesis.

## Abstract 2

Introducing human disease-associated mutations in the membrane skeletal protein spectrin into the nematode Caenorhabditis elegans reveals that spectrin mutations down-regulate ciliary gene expression, affect intraflagellar transport, disrupt axonemal microtubule organization, and inhibit ciliogenesis.

## Introduction

Cilia are evolutionarily conserved organelles that generate forces to drive cell motility and transduce chemical and physical signals to modulate cell behaviors [1–4]. Many ciliary functions are known as mechanical in nature [5], and defects in mechanobiology of cilia lead to ciliopathies, ranging from primary ciliary dyskinesia to polycystic kidney disease to situs inversus [4]. The formation and maintenance of cilia are also affected by mechanical forces. For example, fluid shear disassembles cilia [6] or decreases ciliary length [7–9]; compressive forces can induce the shortening of cilia, and tensile strengths may inhibit cilium formation [10, 11]; and mechanical forces were proposed to function as signals that instruct the formation and orientation of chondrocyte cilia [12]. Despite its importance, we know little about how cells perceive mechanical forces and transduce this information to cilium biogenesis.

The spectrin-based membrane cytoskeleton maintains the structural integrity of the plasma membrane and protects cells from mechanical stresses [13]. Spectrin, actin, and associated proteins form a static polygonal lattice structure underneath the plasma membrane in erythrocyte and many nonerythrocyte cells or an ordered periodic longitudinal array around the circumference of axons and some dendrites [13–16]. The functional unit of spectrin is a rod-shaped tetramer composed of 2 antiparallel heterodimers of α-spectrin and β-spectrin that interact head to head [13]. Mutations in human spectrin genes have been known to impair spectrin-based membrane mechanics, leading to various diseases. The dominant common hereditary elliptocytosis (HE)-associated α-spectrin L260P mutation destabilizes the plasma membrane by locking spectrin in the close dimer conformation [17, 18]. The dominant spinocerebellar ataxia type 5 (SCA5)-associated β-III–spectrin in-frame deletion in the spectrin repeat is predicted to disrupt the overall conformation of the spectrin tetramer and perturbs the localization of the synaptosomal proteins in the cerebellum [19, 20]. Although these studies established the importance of spectrin in neurodegenerative diseases, the underlying cellular basis of pathogenesis is not well understood.

Compared with 2 α-spectrin genes and 5 β-spectrin genes in vertebrate genomes, the nematode *C*. *elegans* genome only contains 1 α-spectrin (*spc-1*), 1 conventional β-spectrin (*unc-70*), and 1 heavy spectrin (βH-spectrin, *sma-1*) gene [21–23], making it an amenable model system to study spectrin-based cell mechanics and to model spectrin-associated human diseases in live animals. Like many molecules essential for mechanobiology such as components in the actomyosin system, the null mutation of α-spectrin (*spc-1*) caused defective embryonic elongation and larval lethality [23], and homozygous null alleles of β-spectrin (*unc-70*) only survived under certain conditions [21], hindering a functional dissection of spectrin in membrane-mechanics–associated events. This work introduced the conserved HE- and spinocerebellar ataxia (SCA)-associated mutations into *C*. *elegans* α- and β-spectrin genes, respectively, and their viable mutant progenies facilitated the demonstration of an essential role of spectrin-based membrane skeleton in ciliogenesis.

## Results

### Generation of disease-associated mutations in *C*. *elegans* spectrin genes

**Fig 1:**
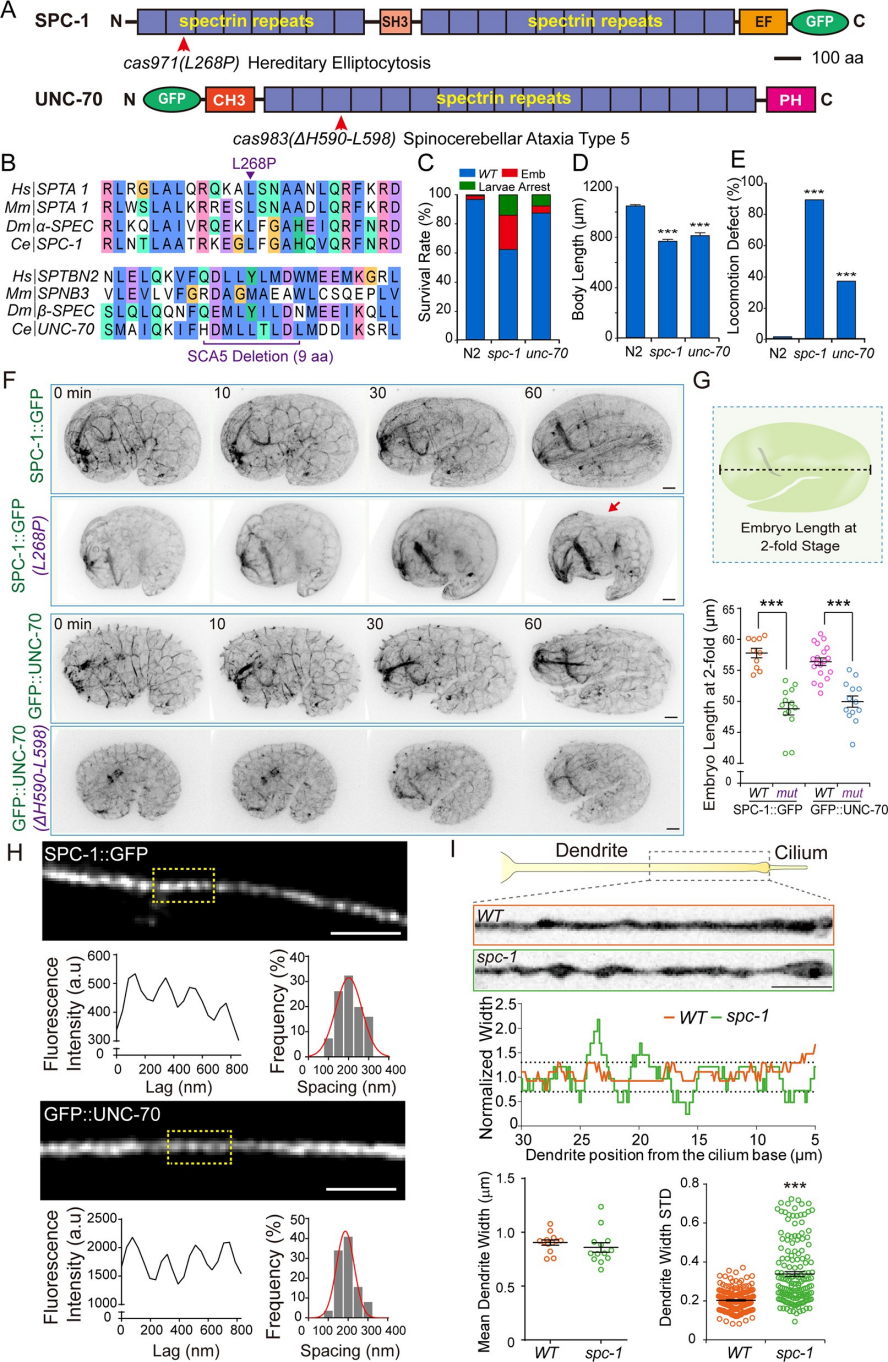
Disease-associated mutations in spectrin impair embryonic elongation and dendrite morphology. (A) Schematic protein domain structure of the *C*. *elegans* alpha-spectrin SPC-1 and beta-spectrin UNC-70. The CRISPR-Cas9–based homologous recombination strategy was used to introduce GFP into the C of SPC-1 or N of UNC-70. Human-diseases–associated mutations (arrows) were introduced into the WT N2 or the GFP KI SPC-1 or UNC-70 animals. Scale bar, 100 amino acids. (B) Alignment of sequences flanking the diseases-associated residue L268P in SPC-1 and an in-frame deletion removing H590-L598 in UNC-70. Both mutations are dominant in human diseases. The heterozygous *C*. *elegans* mutant animals harboring either spectrin mutation caused uncoordinated movement, and this study used homozygous animals for all the phenotype analyses. (C–E) Quantification of embryo survival rates (C), the animal body length (D), and locomotion defect (E) in WT (N2), *spc-1* (*cas971*), and *unc-70* (*cas983*) mutant animals from 3 generations. *N* = 50–100. Error bars are SEM. Comparisons were performed between the WT and mutants, ***p < 0.001. (F–G) Fluorescence inverted images from time-lapse spinning disk confocal movies of SPC-1::GFP, SPC-1 (L260P)::GFP, GFP::UNC-70, and GFP::UNC-70 (SCA5-del) embryos. 0 min, the comma stage; 60 min, the 2-fold stage of WT embryos. The red arrow indicates the deformed dorsal region of the *spc-1* mutant embryo. Embryo lengths are the distance from the embryo head to the tail. (G) Scale bar, 5 μm; comparisons were between the WT and mutants, ***p < 0.001. (H) Representative images of SPC-1::GFP (upper) or GFP::UNC-70 (lower) in neuronal processes of motor neurons in live *C*. *elegans* at the young-adult stage. The low magnification views are in S2C and S2D Fig. The corresponding fluorescence intensity of the boxed region, left; and the histogram of the spacings between periodic structure, right; *N* = 100–150. The red line is a Gaussian fit. Scale bar, 1 μm. (I, upper) A model of the dendrite and cilium in a ciliated sensory neuron of the young-adult *C*. *elegans* (top). A representative image of the boxed region was visualized using a red fluorescent protein Scarlet tagged with a myristoylation signal in WT (middle) and *spc-1* (*cas971*) mutant (bottom) animals. (Middle) Plots of the normalized width along the representative dendrites within 5 to 30 μm from the ciliary base. (Lower) Quantification of the dendrite width and the width SD at each pixel within 5 to 30 μm from the ciliary base in WT or *spc-1* mutant animals. Scale bar, 5 μm. Comparisons were between the WT and mutants, **p < 0.01, ***p < 0.001. Data associated with this figure can be found in S1 Data. a.u, arbitrary unit; C, C terminus; CH3, calponin homology domains type 3; CRISPR-Cas9, clustered regularly interspaced short palindromic repeats-Cas9; EF, EF-hand calcium-binding motifs; Emb, embryo; GFP, green fluorescence protein; KI, knock-in; N, N terminus; PH, Pleckstrin homology domain; SCA-5, spinocerebellar ataxia type 5; SH3, Src homology domain 3; WT, wild type.

Using the CRISPR (clustered regularly interspaced short palindromic repeats)-Cas9-based homologous recombination strategy, we succeeded in replacing L260P in α-spectrin (*spc-1* [*cas971*]) and removed 9 amino acids in the SCA5-associated deletion in β-spectrin (*unc-70* [*cas983*]; Fig 1A and 1B and S1 Fig). Consistent with the reported spectrin-mutant phenotypes [21, 23], both homozygous mutations caused embryonic lethality, larval arrest, and slow growth (Fig 1C); however, 62% of *spc-1* (*cas971*) and 88% of *unc-70* (*cas983*) mutant animals survived and propagated (Fig 1C), which enables the dissection of spectrin-based membrane mechanics throughout animal development. For the viable mutant spectrin animals, the body length was significantly shorter than that of wild-type (WT) N2 strains (Fig 1D), and 89% of *spc-1* (*cas971*) and 38% of *unc-70* (*cas983*) progenies were paralyzed or showed uncoordinated movement (Fig 1E), the motility deficits of which partially mimic the symptoms of SCA [19].

### Spectrin mutations affect embryonic elongation and dendrite shape

To explore the contribution of the *C*. *elegans* spectrin to membrane mechanics, we first visualized the cellular localization of the endogenous spectrin proteins by constructing knock-in (KI) strains to label SPC-1 and UNC-70 with the green fluorescence protein (GFP) at the N or C terminus of spectrin (Fig 1A). The GFP KI strains are indistinguishable from WT animals in growth rates and brood sizes. The endogenous SPC-1 and UNC-70 protein in the KI animals distribute to the cell membrane (S2A and S2B Fig), which is in agreement with the localization pattern observed using immunofluorescence or GFP transgenesis [15, 21, 23, 24]. Recent superresolution imaging studies uncovered that spectrin formed a periodic structure with a spacing of approximately 190 to 200 nm along the axonal and dendritic shafts in various systems including *C*. *elegans* [14–16]. However, it remains unresolved whether the endogenous spectrin adopts the same periodicity in the nervous system of live animals, because the early study relied on an overexpressed β-spectrin GFP reporter [15]. By analyzing the GFP-tagged SPC-1 or UNC-70 in KI worms, we detected a similar periodic structure with a spacing of approximately 190 nm along the axonal shafts (Fig 1H and S2C and S2D Fig), providing strong evidence for the formation of the periodic structure by endogenous spectrin proteins in live animals. Furthermore, we observed a similar spacing structure on the plasma membrane of the epithelial stem cells in *C*. *elegans* larvae (S3 Fig), indicating that the periodicity of spectrin-based membrane skeleton is prevalent in non-neuronal cells.

To examine the cellular effects of both mutations, we knocked the L260P mutation on α-spectrin into the genome of the GFP::SPC-1 KI strain and the 9 aa in-frame deletion into UNC-70::GFP, creating the GFP::SPC-1^L260P^ and UNC-70::GFP^del^ double KI animals. Our live imaging analyses of the early embryonic development in spectrin mutants did not detect any apparent phenotypes for GFP::SPC-1^L260P^ (*N* = 25). We next focused on the process of embryo elongation, during which actomyosin-based mechanic forces convert an embryo from a ball of cells into an elongated 3-fold vermiform shape. Nine out of 39 GFP::SPC-1^L260P^ embryos but none of the GFP::SPC-1 embryos (*N* = 12) failed to complete embryonic elongation, and for the survived GFP::SPC-1^L260P^ embryos, the length was markedly reduced (Fig 1F and 1G). In comparison with the WT embryo, the dorsal portion of the GFP::SPC-1^L260P^ embryo was deformed (red arrow in Fig 1F and S1 and S2 Movies), suggesting that, in the absence of spectrin-based mechanic support, the dorsal cells were likely pulled apart by the actomyosin-based contractile forces. The heavy β-spectrin SMA-1 is responsible for embryonic development, whereas UNC-70 maintains the integrity of axons by regulating cell mechanics in the nervous system [21, 22, 24, 25]. Indeed, we did not detect the failure of embryonic development in UNC-70::GFP^del^ strains; however, the embryo of UNC-70::GFP^del^ was significantly shortened to the same level as the survived GFP::SPC-1^L260P^ embryos (Fig 1F and 1G and S3 and S4 Movies), which correlates with the reduced body length in adult spectrin-mutant animals (Fig 1D).

In the nervous system of spectrin-mutant animals, we found that the dendrite width became less uniform than that of WT dendrites: in some regions, the dendrite was twice wider than that of WT, whereas the dendrite width decreased by half in other areas (Fig 1I). Although the average diameter of dendrite did not change, the SD of the dendrite width markedly increased in spectrin-mutant animals (Fig 1I), unveiling a role of spectrin in maintaining dendrite morphology. Together, these results show that the 2 disease-associated spectrin mutations impair mechanical properties on the plasma membrane and perturb cell shape in *C*. *elegans*.

### Spectrin promotes ciliary gene expression

**Fig 2:**
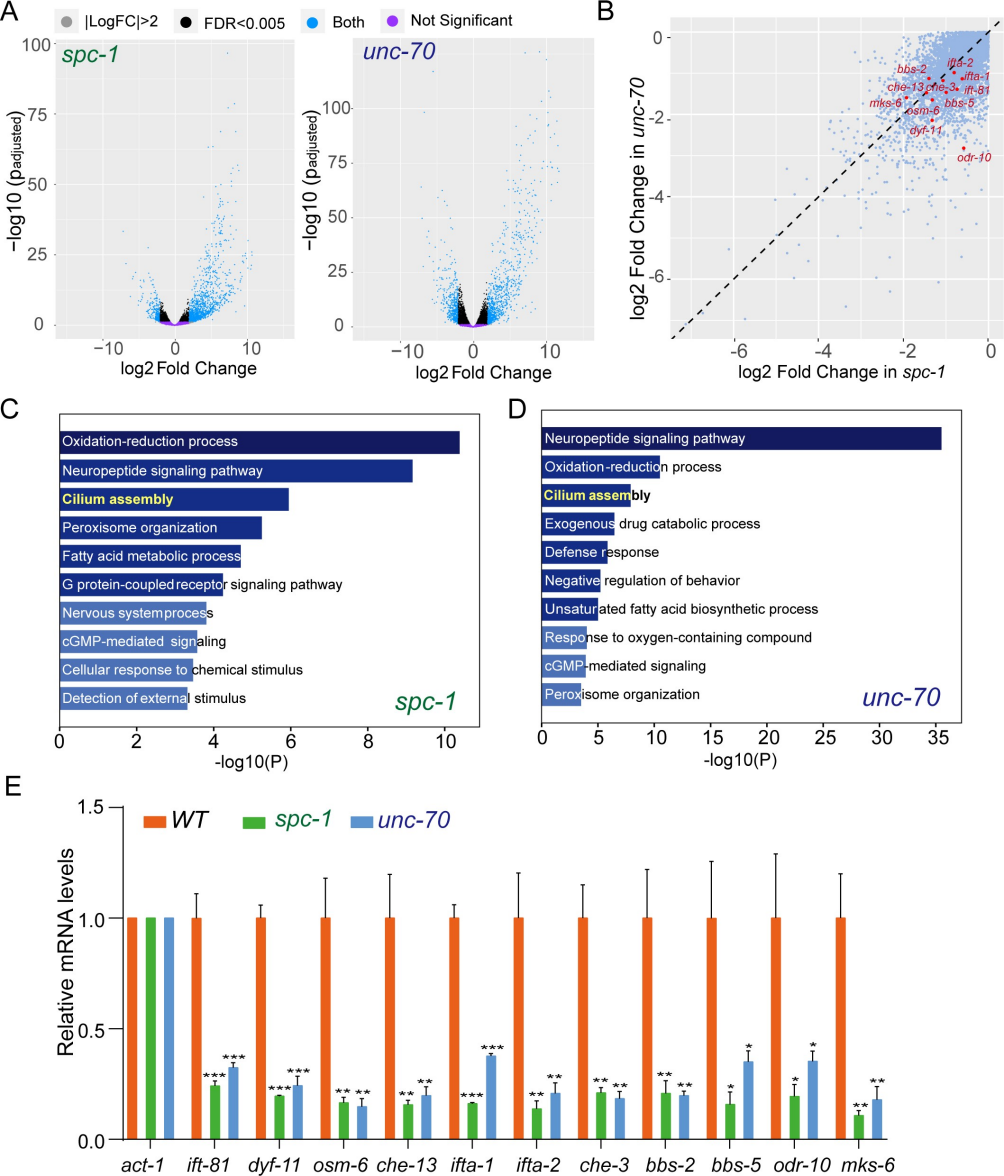
Spectrin promotes ciliary gene expression. (A) Volcano plot of DEGs between WT and *spc-1* or *unc-70* mutant animals. Biological replicates: *N* = 3 for WT, *spc-1*, and *unc-70*. (B) Scatter plot of the gene expression reduction in *spc-1* and *unc-70* mutant animals. Red genes correspond to the significantly down-regulated ciliary genes. (C–D) Gene enrichment analysis for the down-regulated genes in *spc-1* or *unc-70*. Shown are the top 10 most significantly overpresented gene sets. (E) qPCR quantification of the ciliary genes and the control (an actin gene, *act-1*) transcripts in WT and mutants. Three biological replicates for all the genotypes. ***p < 0.001, **p < 0.01, *p < 0.05; one-way ANOVA (and nonparametric). Averages and SDs are plotted. Error bar, SEM. Data associated with this figure can be found in S1 Data. cGMP, cyclic guanosine monophosphate; DEG, differentially expressed gene; FDR, false discovery rate; LogFC, LogFold Change; qPCR, quantitative polymerase chain reaction; WT, wild type.

To obtain comprehensive molecular insights into how spectrin-based mechanics regulate animal development and behaviors, we performed whole transcriptome analyses of WT and spectrin-mutant animals. Measured gene expression by RNA sequencing (RNA-seq) showed a high correlation between *spc-1* and *unc-70* (S4 Fig). We found that 1,714 or 2,067 genes are up-regulated (fold change > 2 and *p <* 0.05) and 1,480 or 1,819 genes are down-regulated (fold change < −2 and *p <* 0.05) in *spc-1* (*cas971*) or *unc-70* (*cas983*) mutant animals, respectively (Fig 2A and S6 and S7 Tables). Among them, 1,089 or 744 genes are up-regulated or down-regulated in both mutants. Intriguingly, gene enrichment analysis revealed that the down-regulated genes in both spectrin mutants are highly enriched in ciliary genes (Fig 2B–2D). Using qPCR (quantitative polymerase chain reaction), we confirmed that a few ciliary transcripts—including intraflagellar transport (IFT) particle A (*ifta-1*, *ifta-2*) and B (*ift-81*, *dyf-11*, *osm-6*, *che-13*) subunits, IFT-dynein heavy (*che-3*), Bardet-Biedl syndrome proteins (*bbs-2*, *bbs-5*), transition zone (TZ) protein (*mks-6*), and ciliary membrane receptor (*odr-10*)—were down-regulated in both spectrin mutants (Fig 2E). These results prompted us to examine whether ciliogenesis was affected by spectrin mutations.

### Spectrin supports ciliogenesis

**Fig 3:**
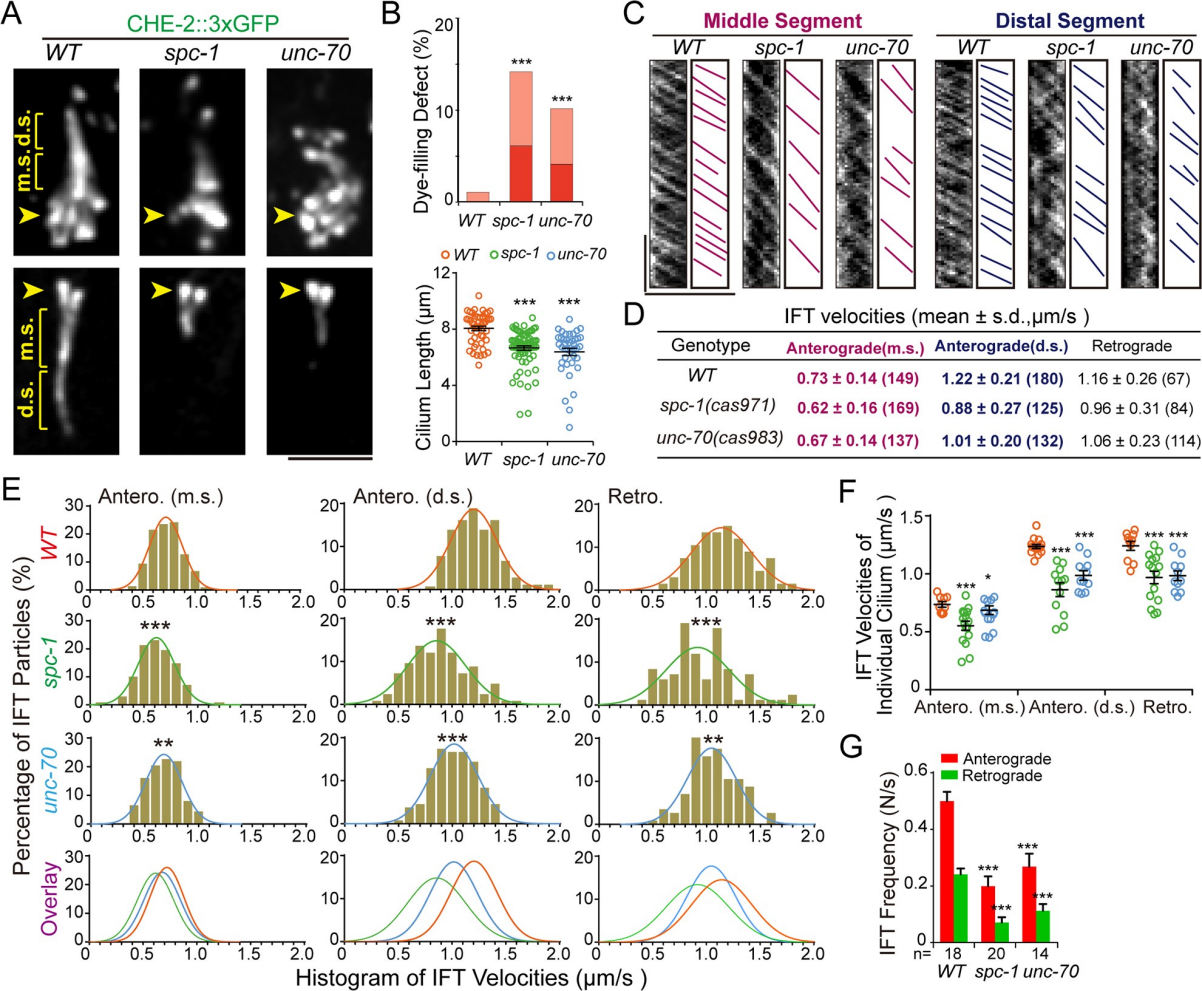
Spectrin supports ciliogenesis. (A) Amphid (top) and phasmid (bottom) cilia in WT, *spc-1* (*cas971*), and *unc-70* (*cas983*) mutants were labeled with IFT80/CHE-2::3×GFP. Arrowheads, the ciliary base and TZ; m.s. or d.s. of sensory cilia. Scale bar, 5 μm. (B, top) Quantifications of the animals showing no staining (red) or weak staining (light red) defects in the dye-filling assay in phasmid cilia from WT and mutant animals (*N* = 250–350). (Bottom) Cilium length (mean ± SEM). The color code for each genotype is indicated. ***p < 0.001. (C) Kymographs show particle movement along the ciliary m.s. or d.s. of phasmid cilia in WT and mutants. Representative particle traces are marked with magenta and indigo lines. The scale bars represent 5 μm (horizontal) and 5 s (vertical). (D) Summary of the anterograde and retrograde velocities of CHE-2::3×GFP in WT and spectrin-mutant cilia that have the d.s. and m.s. Numbers of IFT particles are shown in the brackets. (E) Histogram of CHE-2::3×GFP velocities. (Left) Anterograde IFT along the middle segments. (Middle) Anterograde IFT along the d.s. (Right) retrograde IFT. Each plot was fit by a Gaussian distribution. Comparisons were between the WT and mutants, **p < 0.01; ***p < 0.001. (F) The anterograde and retrograde IFT movement velocities (mean ± SEM) of CHE-2::3×GFP in individual WT and spectrin*-*mutant animals. Color codes for genotype are the same as in (B) and (E). Comparisons were between the WT and mutants, *p < 0.05; ***p < 0.001. (G) Anterograde (red) and retrograde (green) IFT frequencies (mean ± SEM) in WT and spectrin-mutant cilia. Numbers of kymographs used for quantification are shown above the genotypes. Comparisons were between the WT and mutants. ***p < 0.001. Data associated with this figure can be found in S1 Data. d.s., distal segment; GFP, green fluorescence protein; IFT, intraflagellar transport; m.s., middle segment; TZ, transition zone; WT, wild type.

To visualize cilia and IFT, we first genetically crossed the spectrin mutants with the IFT80/CHE-2::3×GFP (3 copies of GFP ()) KI marker, whose transcript level did not change in spectrin mutants. We found that the ciliary length was dramatically reduced from 7.5 μm to 2–3 μm in 13% *spc-1* (*cas971*) or 10% *unc-70* (*cas983*) mutant animals and that IFT was largely reduced in the residual cilia (Fig 3A and 3B and S5 Movie). Consistently, 14% *spc-1* (*cas971*) or 10% *unc-70* (*cas983*) animals failed to or reduced their capacity to uptake the fluorescent lipophilic dye DiI (1,1’-dioctadecyl-3,3,3’,3’,-tetramethylindo-carbocyanine perchlorate) through sensory cilia (Fig 3B), indicating the loss of the ciliary function. For the rest of the spectrin-mutant animals, the ciliary length was comparable to those of WT animals, and the mutant cilia took up the fluorescence dye; however, both anterograde and retrograde IFT along the middle and distal ciliary segments were slowed down, and the IFT frequency also decreased in spectrin-mutant cilia (Fig 3C–3G).

**Fig 4:**
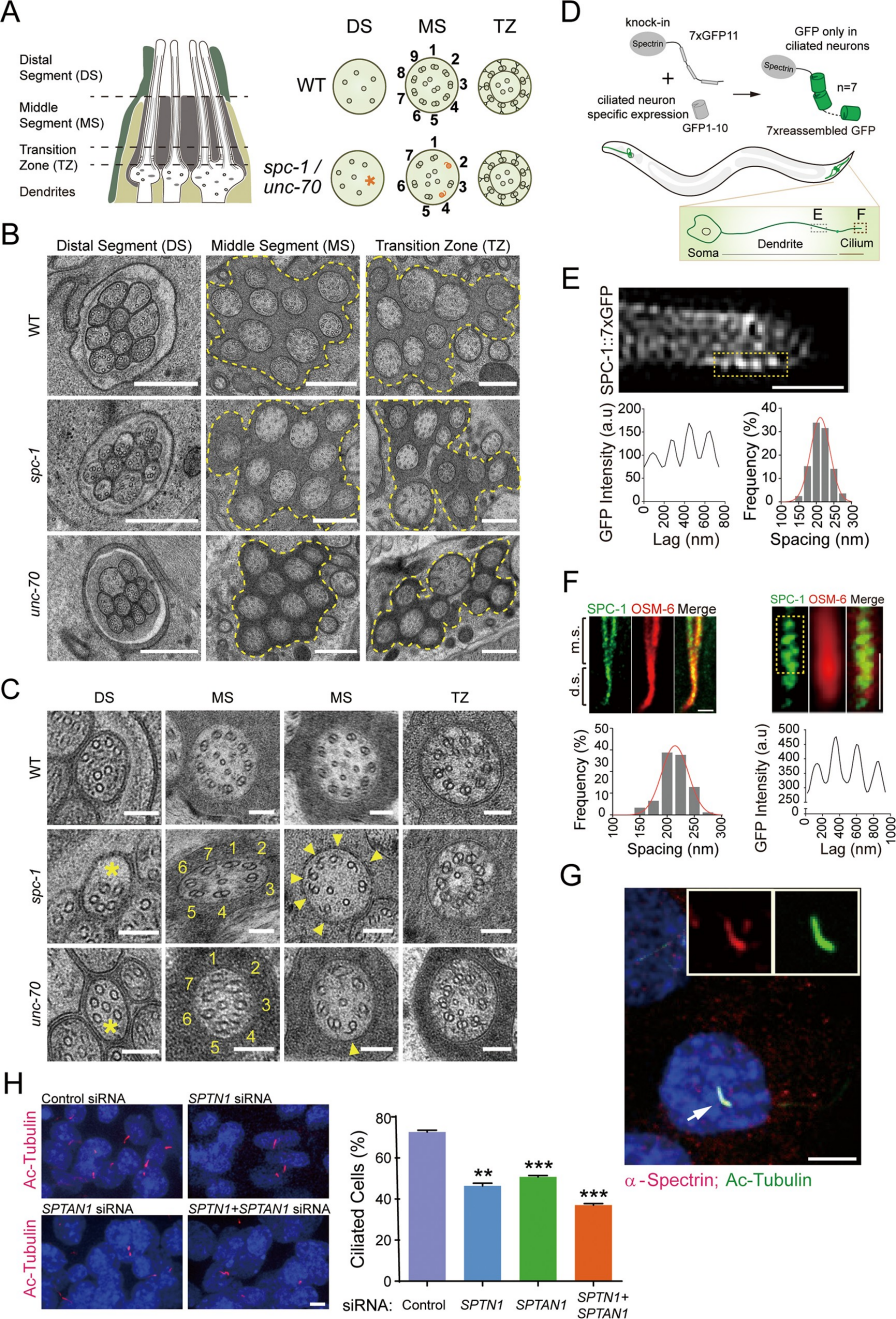
TEM analysis of the ciliary ultrastructure, spectrin localization, and mammalian spectrin in cilia. (A, left) Schematic of the longitudinal ultrastructure of amphid channel cilia (only 4 cilia shown). The glial socket cell (dark green) and sheath cells (light yellow) are shown. (Right) Schematics summarize the traverse ultrastructural phenotypes of cilia from WT (N2), *spc-1* (*cas971*), and *unc-70* (*cas983*) mutant animals. A red asterisk indicates the area lack of axonemal MTs. Incomplete B subfibers are represented in red. (B) Representative TEM images (cross-sections) of the amphid channel cilia (yellow outlines) DS, MS, and TZ in WT and mutants. Scale bar, 500 nm. (C) High magnification images of amphid channel cilia in WT and mutants. Yellow asterisks indicate areas that lack axonemal MTs. Yellow arrowheads indicate incomplete B subfibers in the axoneme. Scale bar, 100 nm. (D, top) Schematic diagram for spectrin fluorescence signal amplification by tandem 7×GFP11 tag, with the specific illumination in *C*. *elegans* ciliated neurons by the split scheme. (Bottom) 7×GFP in ciliated neurons and a zoomed phasmid cilium to show the dendrite and cilium segments used for analyzing spectrin periodic structures in (E–F). (E, top) The representative image of SPC-1::7×GFP in the dendrite of a live *C*. *elegans*. (Bottom) Corresponding fluorescence intensity of the boxed region (left) and the histogram of the spacings between periodic structure (right, *N* = 128 spacings). The red line is a Gaussian fit with a mean of 204.4 nm and an SD of 46.6 nm. Scale bar, 1 μm. (F, top) The representative image of SPC-1::7×GFP (green) and mCherry-tagged OSM-6 (red) in phasmid cilia (left); the magnified image of phasmid cilia (right) and the corresponding SPC-1::7×GFP fluorescence intensity of the boxed region (bottom). Histogram of the spacings between spectrin periodic structure in cilia (*N* = 75 spacings). The red line is a Gaussian fit with a mean of 214.6 nm and an SD of 24.6 nm. Scale bar, 1 μm. (G) Co-localization of mouse α-spectrin and acetylated tubulin immunofluorescence in cilia of IMCD3 cells. DNA was stained with DAPI in blue. (H) α-spectrin (SPTN1 or SPTAN1) knockdown reduced ciliate cell numbers after 48-h siRNA transfection. The number of the cells: control, *N* = 569; RNAi of SPTN1, *N* = 286; SPTAN1, *N* = 402; and SPTN1 + SPTAN1, *N* = 325. Values, mean ± SD (*n* = 3). Student *t* test: **p < 0.01; ***p < 0.001. Scale bars in (G–H), 5 μm. Data associated with this figure can be found in S1 Data. a.u, arbitrary unit; DS, distal segment; GFP, green fluorescence protein; IMCD3, inner medullary collecting duct; MS, middle segment; MT, microtubule; RNAi, RNA interference; siRNA, small interfering RNA; TEM, transmission electron microscope; TZ, transition zone; WT, wild type.

Next, we examined whether the reduced IFT speed and frequency caused any defects in axonemal ultrastructure of the cilia whose length was indistinguishable to that of WT cilia. We performed transmission electron microscopy of high-pressure–frozen, freeze-substituted WT and spectrin-mutant animals (Fig 4A–4C). Our observation from serial sections confirmed that these animals developed the distal and middle ciliary segments as revealed by fluorescence microscopy (Fig 4B), but the organization of axonemal doublet microtubules was defective in *spc-1* (*cas971*) or *unc-70* (*cas983*) mutant cilia. As shown in Fig 4C, in the WT middle ciliary segments, 9 doublets surround a variable number of singlet microtubules, and A tubules from the doublets elongate to the distal ciliary portions, characteristic of immotile cilia as previously described by Perkins and colleagues and Jensen and colleagues [26, 27]. However, 15 out of 35 examined cilia from 3 *spc-1* (*cas971*) mutant animals and 6 out of 21 examined cilia from 2 *unc-70* (*cas983*) worms showed defective axonemal structure: some of the *spc-1* (*cas971*) or *unc-70* (*cas983*) mutant cilia only contained 7 doublets in the middle segments, and some doublets were formed incompletely with an intact A tubule but an incomplete B tubule (Fig 4C). In the distal ciliary parts of spectrin mutants, the microtubule number decreased, and the remaining singlets were unevenly distributed (Fig 4C). In sum, the loss of spectrin inhibited ciliogenesis, affected IFT, and disrupted axonemal microtubule organization.

### Spectrin periodically distributes along *C*. *elegans* sensory cilia

We wondered whether spectrin localizes underneath the ciliary membrane such that spectrin-based membrane mechanics may directly contribute to cilium formation. Examination of spectrin localization to subcellular domains in live animals turns out to be challenging because spectrin expresses in most cell types (S2 Fig), including sensory neurons and their surrounding glial cells, which results in high background fluorescence using either immunofluorescence or GFP KI strategy. To resolve the endogenous spectrin localization in ciliated neurons of *C*. *elegans*, we adopted a three-step methodology that involves genome editing of self-complementing split fluorescent protein and tandem arrangement of the tag (Fig 4D). We first inserted 7 copies of GFP11 tag that encodes the 11th beta-strand of superfolder GFP into the endogenous genomic locus of the alpha-spectrin *spc-1*. Tandem arrangement of 7XGFP11-tags proportionally enhances green fluorescence signal and facilitates high-resolution imaging [28]. Next, we constructed a transgenic animal that expressed GFP1–10 under the control of a ciliated neuron-specific promoter *dyf-1*. After crossing an SPC-1::7XGFP11 KI animal with a P*dyf-1*::GFP1–10 transgenic animal, GFP self-complemented and illuminated spectrin with green fluorescence only in ciliated neurons. As shown in Fig 4F, the endogenous spectrin localized to sensory cilia and the spectrin fluorescence signal arranged into the periodical structures. By projecting of the spectrin fluorescence to the long axis of the cilia, we quantified the distance between the structures and showed that the peak fluorescence separated into a mean value of 214.6 nm and an SD of 24.6 nm, which indicates that spectrin localizes along the cilia with periodicities.

### Mammalian spectrin localizes in cilia and regulates ciliogenesis

To examine whether the contribution of spectrin to ciliogenesis is conserved across species, we performed immunostaining experiments to determine the localization of spectrin in mouse inner medullary collecting duct (IMCD3) cells. Using anti-acetylated α-tubulin immunofluorescence as a cilium marker, we showed that α-spectrin localized in the cilia of IMCD3 cells (Fig 4G). Furthermore, silencing of α-spectrin genes by siRNAs (small interfering RNA) did not cause defects of cell viability or cytoskeleton but significantly reduced ciliate cells numbers (Fig 4H), which indicates an evolutionarily conserved role of spectrin in cilium formation. Spectrin siRNA in mammalian cells or spectrin mutations in *C*. *elegans* generated about 15% penetrance of the ciliary defects (Figs 3B and 4H). The low-penetrance spectrin siRNA in cultured mammalian cells could be due to the heterogeneity of RNAi (RNA interference) treatment, whereas the penetrance and the variance of IFT speeds from the genetically homogenous worm population (Fig 3D and 3E) could be due to the partial loss-of-function alleles of disease-associated point mutation in *spc-1* (*cas971*) or a small in-frame deletion in *unc-70* (*cas983*). Considering that 38% of *spc-1* (*cas971*) and 12% of *unc-70* (*cas983*) mutant embryos did not survive to the larval stage for ciliary phenotype analysis (Fig 1C), the penetrance of ciliary defects in spectrin-mutant worms may be higher than what we detected (Fig 3B).

## Discussion

This work defined an important function of the spectrin-based membrane skeleton in ciliogenesis. Previous studies have discovered the core pieces of machinery that build and maintain cilia and chemical cues that instruct cilium assembly and disassembly [1–3], and our study provides molecular insights into mechanical control of cilium formation. We propose 3 nonexclusive mechanisms to explain how the spectrin-based membrane skeleton can support cilium biogenesis. First, the distribution of spectrin along the ciliary membrane suggests that the spectrin-based membrane mechanics directly regulate the speed and the frequency of IFT, which ferries cargo molecules from the ciliary base to assemble cilia with proper length and ultrastructure. The fact that IFT in the anterograde and retrograde directions are both affected by spectrin mutations (Fig 3C–3G) indicates that mechanic forces systematically impact IFT-motors, as evidenced by optical trap-based manipulations in which applied forces commonly influence the speed, directionality, and processivity of motor proteins [29, 30]. Considering that the OSM-3-kinesin is the only known anterograde IFT motor moving along distal ciliary segments, the reduced IFT velocity in distal segments can be explained by decreasing OSM-3’s speeds (Fig 3C–3E). Spectrin-based mechanic regulation of cilium formation appears to be independent of the actin cytoskeleton because the branched actin network is known to modulate ciliogenesis through vesicular trafficking around the ciliary base but not in cilia [31]. Secondly, dendritic transport of ciliary precursors from the soma to the dendritic endings where sensory cilia assemble must be essential for ciliogenesis. Spectrin exhibits periodical distribution along the dendrite of ciliated neurons (Fig 4E), and the spectrin mutation changes dendrite morphology (Fig 1I), which suggests that spectrin may contribute to cilium assembly by shaping the dendrite and facilitating dendritic transport. Lastly, the spectrin-based membrane skeleton and mechanics regulate ciliogenesis by promoting ciliary gene expression (Fig 2 and S4 Fig and S6 and S7 Tables). The global reduction of ciliary gene expression in spectrin mutants well explains defective IFT in both directions and axoneme phenotypes. Of note, the top category of gene expression reduction in spectrin mutants is the neuropeptide signaling genes (Fig 2C and 2D), which suggests that spectrin-based membrane mechanics may influence an unidentified neuropeptide signaling pathway to promote ciliogenesis. In support of this notion, the glial sheath and socket cells regulate the shape of neuronal receptive endings by modulating the local K(+) and Cl(−) levels [32].

As such, we performed the cell type-specific rescue experiments to determine whether spectrin functions in ciliated neurons or in glial cells to support ciliogenesis. Using a ciliated neuron-specific promoter or glial cell-specific promoters [32] to express spectrin in *spc-1* or *unc-70* mutant animals, we showed that the expression of spectrin from ciliated neurons but not from glial cells rescued ciliary defects (S5 Fig). These data revealed a cell-autonomous contribution of spectrin to ciliogenesis and are consistent with the observation that knockdown of spectrin by siRNA caused defective cilium assembly in the cultured mammalian IMCD3 cells (Fig 4H). Considering that the glial socket cell appears expanded and accumulates vesicles in spectrin-mutant animals (S6 Fig), the negative rescue results from glial cells may not exclude the function of glial spectrin in ciliogenesis.

Although spectrin is best known for its mechanical support for the plasma membrane in a variety of eukaryotic cells, a spectrin-based cytoskeleton is associated with intracellular membrane organelles such as the Golgi apparatus, in which spectrin contributes to its structural integrity and secretory activity [33]. A recent study showed that extracellular physical cues reverberate Golgi mechanics and control lipid metabolism [34], and it will be interesting to examine whether spectrin is involved. Interestingly, axons have been recently shown a reversible strain-softening response, which originates from the axonal membrane skeleton comprising actin filaments and spectrin proteins [35]. Perturbations of this mechanism cause axonal beading [36], similar to the irregular structure of the dendrites in spectrin-mutant worms (Fig 1I). Hence, the spectrin-based membrane skeleton appears to support a broad spectrum of the membrane systems ranging from the axonal and dendritic plasma membrane, endomembranes to cilia. The changes of cell mechanics alter gene expression and cell fates through the transcription factors YAP (yes-associated protein) and TAZ (transcriptional coactivator with PDZ-binding motif) in the Hippo signaling pathway in physiology and disease conditions [37]. The *C*. *elegans* Hippo components are essential for neuroblast division and migration but not ciliogenesis [38]. Future studies will be needed to elucidate the molecular pathways of spectrin-based membrane mechanics implicated in ciliary gene expression and IFT, which will provide an entry point for understanding how mechanic forces modulate organelle biogenesis.

Our findings that mammalian spectrin localizes in cilia and promotes ciliogenesis (Fig 4G and 4H) support the connection between cilia and cerebellar ataxia and provide insights into SCA5 pathogenesis. Genetic deficiencies that are associated with ciliopathies including Bardet-Biedl syndrome, Joubert syndrome, Meckel-Gruber syndrome, and Orofaciodigital syndrome have been implicated in congenital disorders with cerebellar abnormalities [4, 39]. The SCA protein ataxin 10 interacts with the ciliary proteins NPHP5 (nephronophthisis 5) and CEP290 (centrosomal protein 290), and a patient with Joubert syndrome had a mutation of ataxin 10 itself [40]. Mutations in the tau tubulin kinase 2, which are crucial for initiating ciliogenesis at the basal body, caused SCA type 11 [41]. Future studies can be productive to examine whether the ciliary structure or function are defective in Purkinje cells and granule cell progenitors from the cerebellum with HE and SCA5. On the other hand, several SCAs exhibit ciliopathy-associated syndromes such as retinal degeneration [4, 39], and our results uncover the ciliary defects in spectrin mutants, raising the possibility that SCA5 deletions or other spectrin mutations may be the unidentified molecular lesions that are associated with ciliary diseases. We anticipate that the restoration of ciliary function in the cerebellum may protect from neurodegenerative disorders and that manipulation of mechanobiology of cilium can be an effective therapeutic strategy for ciliopathies.

## Materials and methods

### *C*. *elegans* strains and genetics

*C*. *elegans* strains were raised on NGM plates seeded with *Escherichia coli* strain OP50 at 20°C. S1–S5 Tables summarize the primers, plasmids, and strains used in this study.

### Molecular biology

CRISPR-Cas9 targets were inserted to the pDD162 vector (Addgene #47549) by linearizing this vector with primers listed in S1 Table. The resulting PCR products containing 15 base pairs (bp) overlapped double-strand DNA ends were treated with DpnI digestion overnight and transformed into *E*. *coli*. The linearized PCR products were cyclized to generate plasmids by spontaneous recombination in bacteria. For fluorescence tag KI (S7 Fig), homology recombination (HR) templates were constructed by cloning the 2 kb 5ʹ and 3ʹ homology arms into pPD95.77 plasmids using In-Fusion Advantage PCR cloning kit (Clontech, Palo Alto, CA, USA, Cat. # 639621). We used the CRISPR design tool (http://crispr.mit.edu) to select the target sequence. GFP and 7×GFP11 tag were added to the C terminus of SPC-1, whereas UNC-70 was tagged with an N-terminal GFP. We generated SPC-1-L268P and UNC-70-ΔH590-L598 mutation KIs (S7 Fig) in the SPC-1::GFP and GFP::UNC-70 KI background by using similar cloning strategies.

### RNA extraction, library preparation, and sequencing

Total RNA was extracted from L1 worms with TRIzol (Invitrogen, Carlsbad, CA, USA) according to the manufacturer's protocol. The RNA quality was assessed on the Agilent Bioanalyzer 2100 system. The samples with RIN > 6 were processed; 50 ng to 500 ng of total RNA was used for library preparation using the KAPA RNA HyperPrep Kit (KAPA Biosystems, Wilmington, MA, USA). Library samples were sequenced on an Illumina HiSeq platform. A total of 150-bp paired-end reads were generated.

### RNA-seq data processing

Raw sequencing reads were first trimmed using TrimGalore (version 0.4.4) to remove the low-quality bases and adaptor sequences. After trimming, paired-end reads with at least 20 nucleotides in length were aligned to *C*. *elegans* reference genome (ce10) using STAR (2.5.4b) and quantified by HTSeq (version 0.9.1). Only uniquely mapped reads were used to calculate the relative expression level of the gene. Differentially expressed genes (DEGs) were calculated using DESeq2 with adjusted *p* ≤ 0.05 and log2 fold change ≥ 1. Metascape was used to identify gene enrichment terms in up- or down-regulated genes. S6 and S7 Tables summarize RNA-seq data.

### Reverse transcription and quantitative real-time PCR

cDNA was synthesized in a 20 μl reaction volume using the RevertAid First Strand cDNA Synthesis Kit (Thermo Fisher Scientific, Waltham, MA, USA); 2 μl of 1:4 dilution of cDNA was used as the template in a 20 μl reaction volume from the FastStart Universal SYBR Green Master (Roche Diagnostics, Basel, Switzerland). A list of the real-time PCR primers is in the resource S2 Table. Quantitative real-time PCR was performed in 3 replicates using the Bio-Rad CFX96 Real-Time System. Data were analyzed using the standard curve method. The experiments were repeated 3 times on independent RNA preparations.

### Dye-filling assay

Young-adult worms were randomly collected into 200 μl M9 solution and mixed with equal volume dyes (DiI, 1,1’-dioctadecyl-3,3,3’,3’,-tetramethylindo-carbocyanine perchlorate; Sigma-Aldrich, St. Louis, MO, USA) at working concentration (20 μg/ml), followed by incubation at room temperature in the dark for 30 min. Worms were transferred to seeded NGM plates and examined for dye uptake 1 h later using a fluorescence stereoscope. At least 100 worms of each strain were examined in 2 independent assays.

### Live-cell imaging

Young-adult worms were anesthetized with 0.1 mmol/L levamisole in M9 buffer and mounted on 3% agarose pads at 20°C. Live-cell imaging was performed on an Axio Observer Z1 microscope (Carl Zeiss, Oberkochen, Germany) equipped with a 100×, 1.49 numerical aperture (NA) objective, an electron-multiplying (EM) charge-coupled device (CCD) camera (Andor iXon+ DU-897D-C00-#BV-500), and the 488 nm and 561 nm lines of a Sapphire CW CDRH USB Laser System attached to a spinning disk confocal scan head (Yokogawa CSU-X1 Spinning Disk Unit). Our high-resolution live imaging system includes an Olympus IX83 microscope equipped with a 150×, 1.45 NA objective lens, a Neo 5.5 sCMOS Camera (DC-152Q-C00-FI; Andor Technology), and the same spinning disk confocal modules as mentioned above. Time-lapse images were acquired by μManager (https://www.micro-manager.org) at an exposure time of 200 ms. Images of spectrin periodicity were collected on a Nikon (Tokyo, Japan) A1R laser-scanning confocal microscope with a CFI Plan Apo 100× oil immersion objective (NA 1.45) and 488-nm lasers with 150 nm X-Y resolution or a Hessian matrix-based structure illumination microscopy with 88 nm X-Y resolution.

### Transmission electron microscopy

Adult worms were loaded onto a 50-μm–thick aluminum specimen carrier and rapidly frozen with a Leica EM HPM100 high-pressure freezing system(Leica Microsystems GmbH, Wetzlar, Germany). After freezing, carriers were transferred into 2 ml microcentrifuge tube containing 1 ml acetone solution of 1% osmium tetroxide and 0.1% uranyl acetate (Ted Pella, Inc., Redding, CA, USA) under liquid nitrogen. The tubes were then placed in Leica EM AFS2 machine(Leica Microsystems GmbH, Wetzlar, Germany) and processed using standard FSF program: −90°C for 48 h, −60°C for 24 h, −30°C for 18 h, and finally to 4°C. Freeze-substituted fixed specimens were washed 3 times with pure acetone and infiltrated with SPI-PON 812 resin(SPI Supplies, West Chester, PA, USA). The specimens were subsequently embedded in a flat mold and polymerized at 60°C; 90 nm sections were obtained with a Leica EM UC7 Ultramicrotome (Leica Microsystems GmbH, Wetzlar, Germany) and picked on 200 mesh copper grids. Sections were poststained with 2% uranyl acetate and Reynold’s lead citrate to enhance contrast and imaged on an FEI Tecnai G2 Spirit (120 kV) electron microscope (FEI Company, Hillsboro, OR, USA).

### Cell culture and transfection

IMCD3 cells were maintained at 37°C in 5% CO_2_ in air in DMEM/F12 (Invitrogen, Carlsbad, CA, USA) containing 10% fetal bovine serum (FBS), 0.3 mg/ml glutamine (Sigma-Aldrich, St. Louis, MO, USA), 100 U/ml penicillin (Invitrogen, Carlsbad, CA, USA), and 100 U/ml streptomycin (Invitrogen, Carlsbad, CA, USA). To induce ciliogenesis, cells were shifted from 10% serum to 0.05% serum for 24 h. Plasmids were transfected into cells using Lipofectamine 3000 (Invitrogen, Carlsbad, CA, USA) following the manufacturer’s instructions. Cells were shifted from 10% serum to 0.05% serum for 48 h after transfection of shRNA to induce ciliogenesis before fixation. The target and control scrambled sequences were from the mouse shRNA library of Sigma. The control scrambled sequence was 5ʹ-CAACAAGATGAAGAGCACCAA-3ʹ. The alpha-spectrin Spta1 and Sptan1 shRNAs were a pool of 3 different plasmids, targeting the Spta1 sequence: 5ʹ-CCCACTAACATTCAGAGGAAA-3ʹ, 5ʹ-CCACCGTAACAGACGTGAAAT-3ʹ, 5ʹ-GCAGATCAAGAACTTTGAAAT-3ʹ, and targeting the Sptan1 sequence: 5ʹ-GCAGCAACAATTTAATCGAAA-3ʹ, 5ʹ-GCATCCACTAACAGAGGCAAA-3ʹ, 5ʹ-GCACACGAAGTACAGAGGTTT-3ʹ.

### Immunofluorescence staining

Cells for anti-acetylated tubulin staining were incubated on ice for 30 min before fixation to depolymerize cytoskeletal microtubules. Cells were fixed with 4% paraformaldehyde (PFA) for 15 min at room temperature. Cells were then washed 3 times with PBS and permeabilized in 0.5% Triton X-100 in PBS for 15 min. Cells were blocked in 4% bovine serum albumin (BSA) for 1 h at room temperature and incubated with primary antibodies at 4°C overnight. Primary antibodies used for immunofluorescence are mouse anti-acetylated-tubulin (1:1,000, Sigma-Aldrich, St. Louis, MO, USA, Cat. #T6793) and rabbit anti-SPTA1 (alpha spectrin, 1:100, ABclonal Technology, Boston, MA, USA, Cat. #A12355). Alexa 488- or Alexa 549-conjugated secondary antibodies (Abbkine, Woburn, MA, USA) were applied for 1 h at room temperature. DNA was stained with DAPI. Secondary antibodies used for immunofluorescence were Dylight 488, Goat Anti-Rabbit IgG (1:500, Abbkine, Woburn, MA, USA, Cat. #A23220-1); Dylight 549, Goat Anti-Rabbit IgG (1:500, Abbkine, Woburn, MA, USA, Cat. #A23320-1); Dylight 488, Goat Anti-Mouse IgG (1:500, Abbkine, Woburn, MA, USA, Cat. #A23210-1); and Dylight 549, Goat Anti-Mouse IgG (1:500, Abbkine, Woburn, MA, USA, Cat. #A23310-1).

### Quantifications and statistical analysis

ImageJ software was used to circumscribe the fluorescence field and to measure the fluorescence intensity. We used the two-tailed Student *t* test analysis to determine the statistical differences as indicated in figure legends.

### Construction of spectrin transgene and transformation

P*dyf-1*::*spc-1* and P*dyf-1*::*unc-70* constructs were generated using a PCR-fusion–based approach by placing a 454 bp ciliated neuron-specific promoter of *dyf-1* adjacent to a DNA fragment containing *spc-1* or *unc-70* and its 3ʹ UTR amplified from the *C*. *elegans* genomic DNA. P*itr-1*::*spc-1* and P*itr-1*::*unc-70* constructs were generated using the same PCR-fusion–based approach by placing a 2,335 bp socket cell-specific promoter of *itr-1* adjacent to a DNA fragment containing *spc-1* or *unc-70* and its 3ʹ UTR amplified from genomic DNA. P*vap-1*::*spc-1* and P*vap-1*::*unc-70* constructs were generated using a PCR-fusion–based approach by placing a 5,205 bp sheath cell-specific promoter of *vap-1* adjacent to a DNA fragment containing *spc-1* or *unc-70* and its 3ʹ UTR amplified from genomic DNA. Transgenic lines carrying extrachromosomal arrays of the *spc-1* or *unc-70* expression constructs were generated by germline transformation with the selection marker P*egl-17*::*mCherry-Myri* and P*egl-17*::*mCherry*::*TEV-S*::*his-24* into homozygous *spc-1* (*cas971*) or *unc-70* (*cas983*) mutant animals.

## Supporting information

S1 Data Data underlying figures and Supporting information figures. Data for Fig 1C–1E and 1G–1I; Fig 2E; Fig 3B and 3E–3G; Fig 4E, 4F and 4H; S3C and S3D Fig; and S5 Fig.
(XLSX)

S1 Fig Sanger sequencing chromatograms of *spc-1* and *unc-70* mutants. Disease-associated *spc-1* (*L268P*) (A) and *unc-70* (ΔH590-L598) (B) mutations were confirmed by Sanger sequencing. The sequence of the engineered *C*. *elegans* genomic DNA containing the mutation is shown with the corresponding control sequence below. Arrows in the chromatogram indicate the site of the mutation. Red rectangles in the control sequence indicate the affected amino acids.
(TIF)

S2 Fig Spectrin distribution in *C. elegans*. (A–B) The alpha-spectrin SPC-1 (A) and beta-spectrin UNC-70 (B) in *C*. *elegans* development. The GFP KI embryos at the 1-cell stage, the 8-cell stage, comma stage, and the larva at L1 (upper right) or L4 (lower) stage were imaged. Scale bars, 5 μm in the upper panel and 50 μm in the lower panel. (C–D) The representative images of SPC-1 (C) and UNC-70 (D) from GFP KI animals. High magnification images of the selected regions in the yellow dotted boxes are shown in Fig 1H. Scale bars, 5 μm. GFP, green fluorescence protein; KI, knock-in.
(TIF)

S3 Fig SPC-1 and UNC-70 exhibit periodic patterns in the *C. elegans* epithelial stem cells/seam cells. (A, left) A representative still image of SPC-1::GFP KI animal showing the distribution in neurites and membrane. (Right) Representative image of the seam cells from a double-labeled animal showing UNC-70 (green, middle) and SPC-1 (red, bottom) co-localization (top). Scale bars, 5 μm. (B) Representative images of SPC-1::GFP (upper) or GFP::UNC-70 (lower) from the plasma membrane of seam cells in live *C*. *elegans* (top). Scale bar, 1 μm. (C) Histogram of the spacings between adjacent of SPC-1::GFP (left) or GFP::UNC-70 (right) structures (*N* = 100–150 spacings), and the red line is a Gaussian fit. Data associated with this figure can be found in S1 Data. GFP, green fluorescence protein; KI, knock-in.
(TIF)

S4 Fig Scatterplots depict the correlation of the RNA levels measured by RNA-seq between *spc-1* and *unc-70*. (TIF)

S5 Fig Defects in the dye-filling assay from WT, mutant, and rescue animals. Quantifications of the animals showing no staining (red) or weak staining (light red) defects in the dye-filling assay from WT, mutant animals, or their rescue animals (*N* = 117–273). Statistical significances between underlined pairs are based on Student *t* test, ***p < 0.001. Data associated with this figure can be found in S1 Data. n.s., not significant; WT, wild type.
(TIF)

S6 Fig TEM images (cross-sections) of the amphid channel ciliary distal segments. Yellow arrows indicate the boundary of socket cells. White asterisks indicate that vesicles accumulated in socket cells. TEM, transmission electron microscope.
(TIF)

S7 Fig Generation of KI *C. elegans* and HR templates with disease-related mutations. (A) Schematic design of the CRISPR-Cas9–assisted KI for *C*. *elegans*. sgRNAs targeting the 5ʹ or 3ʹ ends of genes and approximately 1 kb homology arms and fluorescent tags were inserted into HR templates. (B) Summary of CRISPR targets, homology arms, and tags. (C) Construction of HR templates to generate disease-related mutation KI strains. CRISPR targets and PAM motifs in WT genomes are colored in red and green, respectively. Disease-associated *spc-1* (*L268P*) and *unc-70* (ΔH590-L598) mutations in templates are colored in blue. CRISPR-Cas9, clustered regularly interspaced short palindromic repeats- Cas9; HR, homology recombination; KI, knock-in; PAM, protospacer adjacent motif; sgRNA, single-guide RNA; WT, wild type.
(TIF)

S1 Movie The embryonic development of SPC-1::GFP KI animal. Inverted fluorescence time-lapse movie of SPC-1::GFP embryo. Frames were taken every 2 min. The display rate is 7 frames per second. GFP, green fluorescence protein; KI, knock-in.
(AVI)

S2 Movie The embryonic development of SPC-1 (L268P)::GFP KI animal. Inverted fluorescence time-lapse movie of SPC-1 (L268P)::GFP embryo. Frames were taken every 2 min. The display rate is 7 frames per second. GFP, green fluorescence protein; KI, knock-in.
(AVI)

S3 Movie The embryonic development of GFP::UNC-70 KI animal. Inverted fluorescence time-lapse movie of GFP KI GFP::UNC-70 embryo. Frames were taken every 2 min. The display rate is 7 frames per second. GFP, green fluorescence protein; KI, knock-in.
(AVI)

S4 Movie The embryonic development of GFP::UNC-70 (ΔH590-L598) KI animal. Inverted fluorescence time-lapse movie of GFP::UNC-70 (ΔH590-L598) embryo. Frames were taken every 2 min. The display rate is 7 frames per second. GFP, green fluorescence protein; KI, knock-in.
(AVI)

S5 Movie IFT of GFP::CHE-2 in the phasmid cilia of WT (left), *spc-1* (middle), and *unc-70* (right) mutant animals. Images were taken using a time-lapse fluorescence microscope attached to a spinning disk confocal scan head (CSU-X1 Spinning Disk Unit; Yokogawa Electric Corporation). Frames were taken at an interval of 200 ms. The display rate was 20 frames per second. Scale bar: 5 μm. GFP, green fluorescence protein; IFT, intraflagellar transport; WT, wild type.
(AVI)

S1 Table Targets of CRISPR and primers for molecular analysis. CRISPR, clustered regularly interspaced short palindromic repeats.
(DOCX)

S2 Table Oligonucleotides for qPCR. qPCR, quantitative polymerase chain reaction.
(DOCX)

S3 Table Primers and plasmids used for plasmid cloning in this study. (DOCX)

S4 Table Primers for SOEing PCR. SOE, splicing by overlapping extension.
(DOCX)

S5 Table *C. elegans* strains used in this study. (DOCX)

S6 Table Summary table of RNA-seq data (*spc-1*), related to Fig 2. RNA-seq, RNA sequencing.
(XLSX)

S7 Table Summary table of RNA-seq data (*unc-70*), related to Fig 2. RNA-seq, RNA sequencing.
(XLSX)

## Abbreviations

a.u: arbitrary unit
bp: base pairs
BSA: bovine serum albumin
CCD: charge-coupled device
CEP290: centrosomal protein 290
CH3: calponin homology domains type 3
CRISPR-Cas9: clustered regularly interspaced short palindromic repeats- Cas9
DEG: differentially expressed gene
DiI: 1,1’-dioctadecyl-3,3,3’,3’,-tetramethylindo-carbocyanine perchlorate
EF: EF-hand calcium-binding motifs
EM: electron-multiplying
FBS: fetal bovine serum
GFP: green fluorescence protein
HE: hereditary elliptocytosis
HR: homology recombination
IFT: intraflagellar transport
IMCD3: inner medullary collecting duct
KI: knock-in
MT: microtubule
NA: numerical aperture
NPHP5: nephronophthisis 5
PFA: paraformaldehyde
PH: Pleckstrin homology domain
qPCR: quantitative polymerase chain reaction
RNAi: RNA interference
RNA-seq: RNA sequencing
SCA: spinocerebellar ataxia
SCA5: spinocerebellar ataxia type 5
SH3: Src homology domain 3
siRNA: small interfering RNA
TAZ: transcriptional coactivator with PDZ-binding motif
TEM: transmission electron microscope
TZ: transition zone
WT: wild type
YAP: Yes-associated protein
3×GFP: 3 copies of GFP
