# Supplementary figures and images for: Spectrin-based membrane skeleton supports ciliogenesis
Source: PLoS Biol. 2019 Jul 12;17(7):e3000369. doi: 10.1371/journal.pbio.3000369 (PMC6655744; doi:10.1371/journal.pbio.3000369)

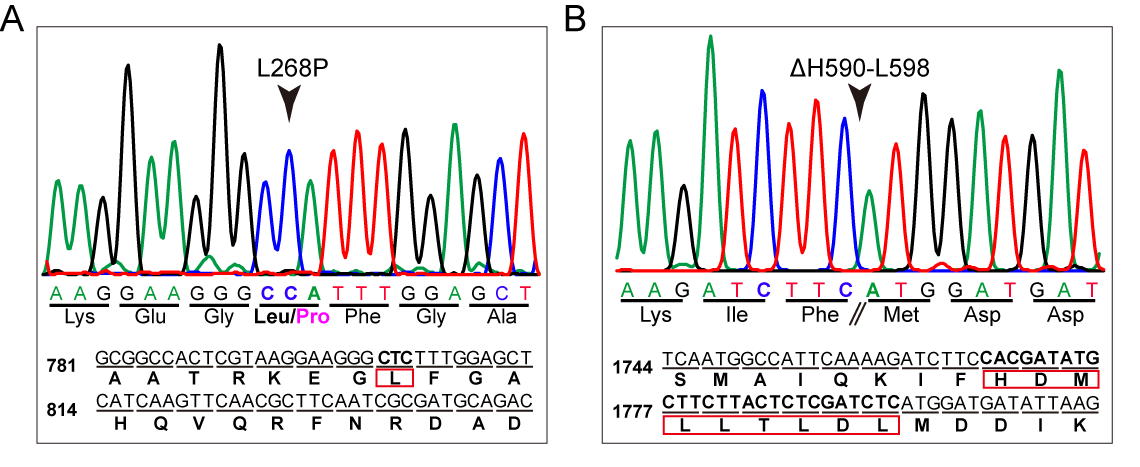

Supplement: S1 Fig — Disease-associated spc-1 (L268P) (A) and unc-70 (ΔH590-L598) (B) mutations were confirmed by Sanger sequencing. The sequence of the engineered C. elegans genomic DNA containing the mutation is shown with the corresponding control sequence below. Arrows in the chromatogram indicate the site of the mutation. Red rectangles in the control sequence indicate the affected amino acids. (TIF) [file pbio.3000369.s002.tif]

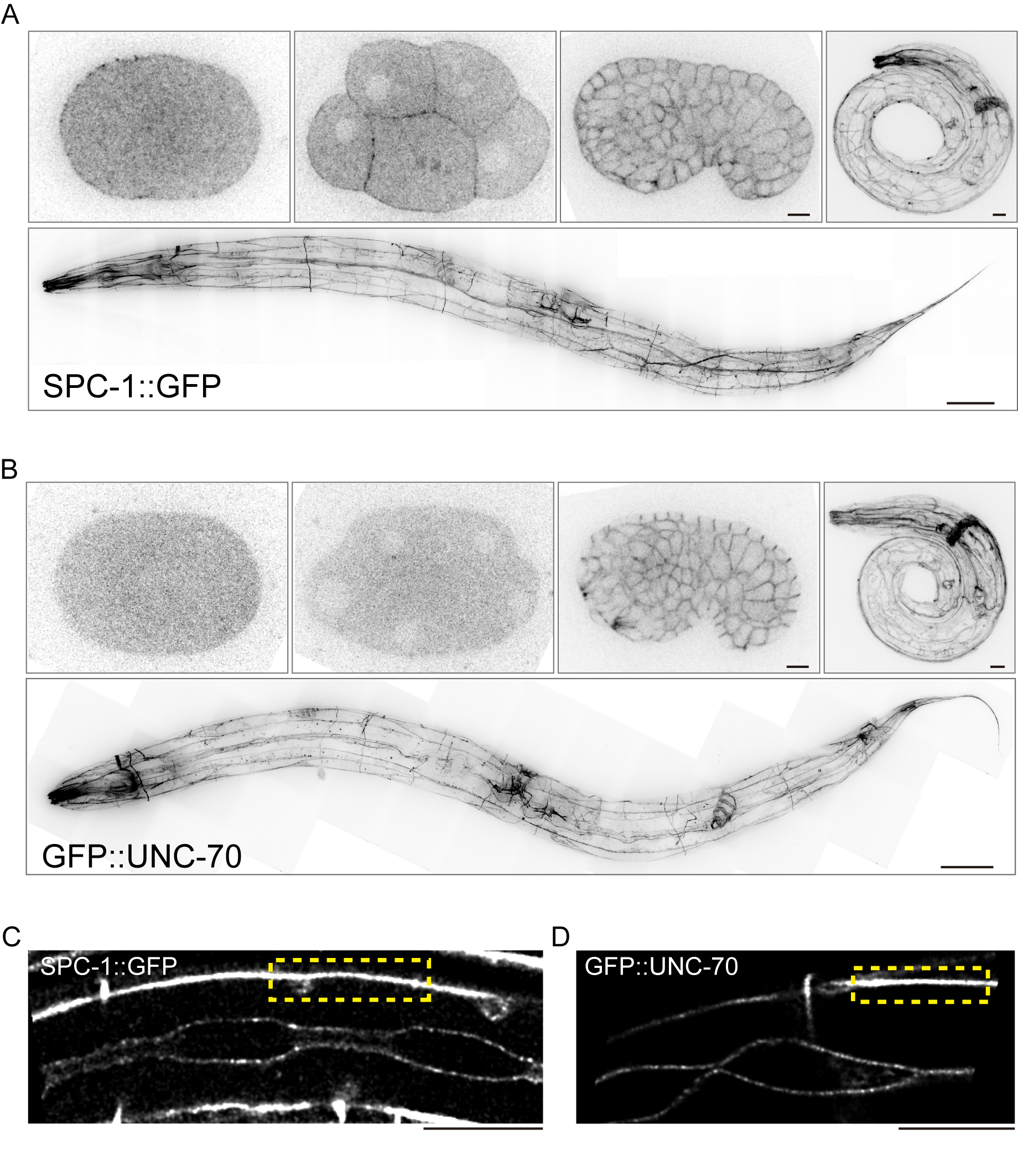

Supplement: S2 Fig — (A–B) The alpha-spectrin SPC-1 (A) and beta-spectrin UNC-70 (B) in C. elegans development. The GFP KI embryos at the 1-cell stage, the 8-cell stage, comma stage, and the larva at L1 (upper right) or L4 (lower) stage were imaged. Scale bars, 5 μm in the upper panel and 50 μm in the lower panel. (C–D) The representative images of SPC-1 (C) and UNC-70 (D) from GFP KI animals. High magnification images of the selected regions in the yellow dotted boxes are shown in Fig 1H. Scale bars, 5 μm. GFP, green fluorescence protein; KI, knock-in. (TIF) [file pbio.3000369.s003.tif]

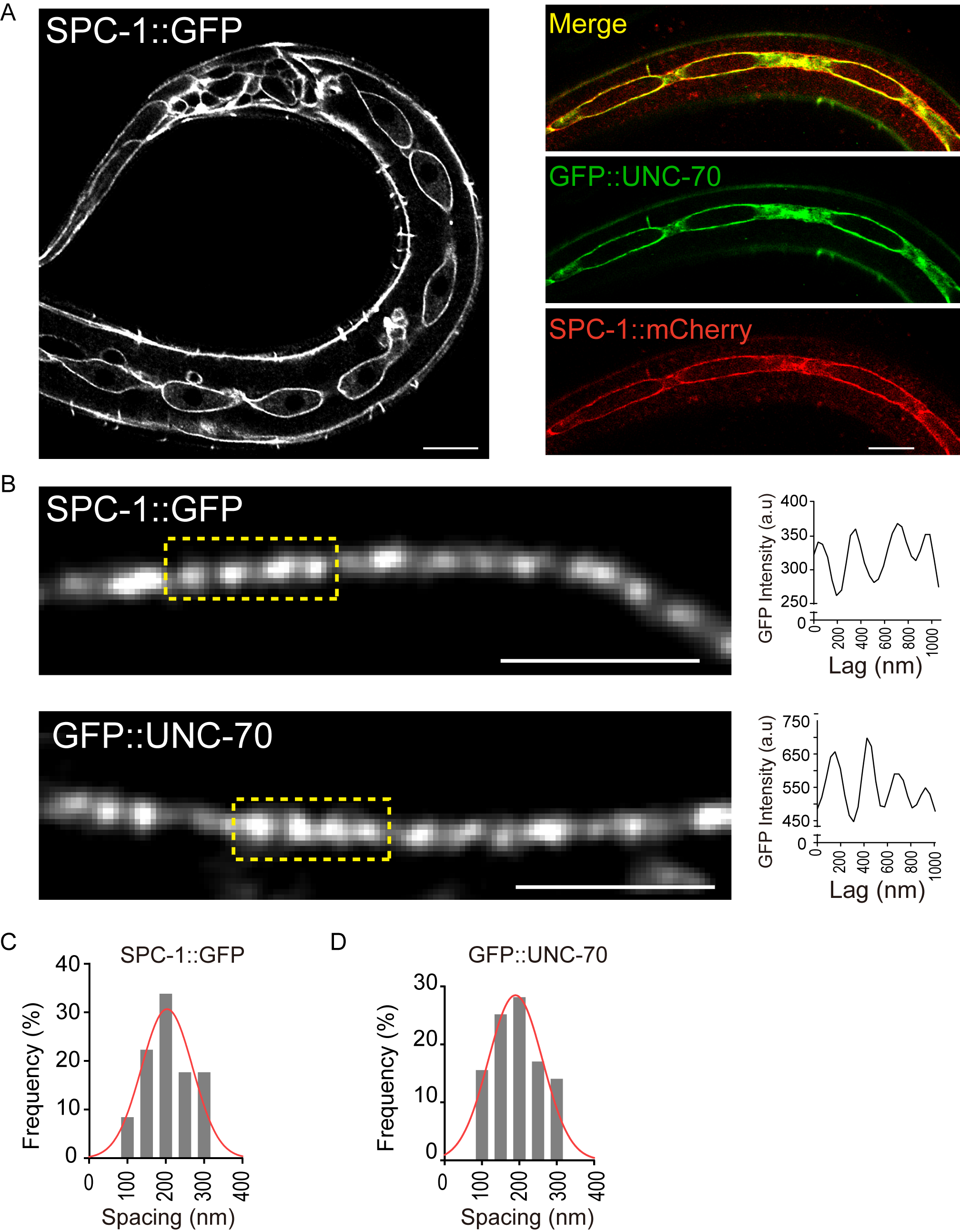

Supplement: S3 Fig — (A, left) A representative still image of SPC-1::GFP KI animal showing the distribution in neurites and membrane. (Right) Representative image of the seam cells from a double-labeled animal showing UNC-70 (green, middle) and SPC-1 (red, bottom) co-localization (top). Scale bars, 5 μm. (B) Representative images of SPC-1::GFP (upper) or GFP::UNC-70 (lower) from the plasma membrane of seam cells in live C. elegans (top). Scale bar, 1 μm. (C) Histogram of the spacings between adjacent of SPC-1::GFP (left) or GFP::UNC-70 (right) structures (N = 100–150 spacings), and the red line is a Gaussian fit. Data associated with this figure can be found in S1 Data. GFP, green fluorescence protein; KI, knock-in. (TIF) [file pbio.3000369.s004.tif]

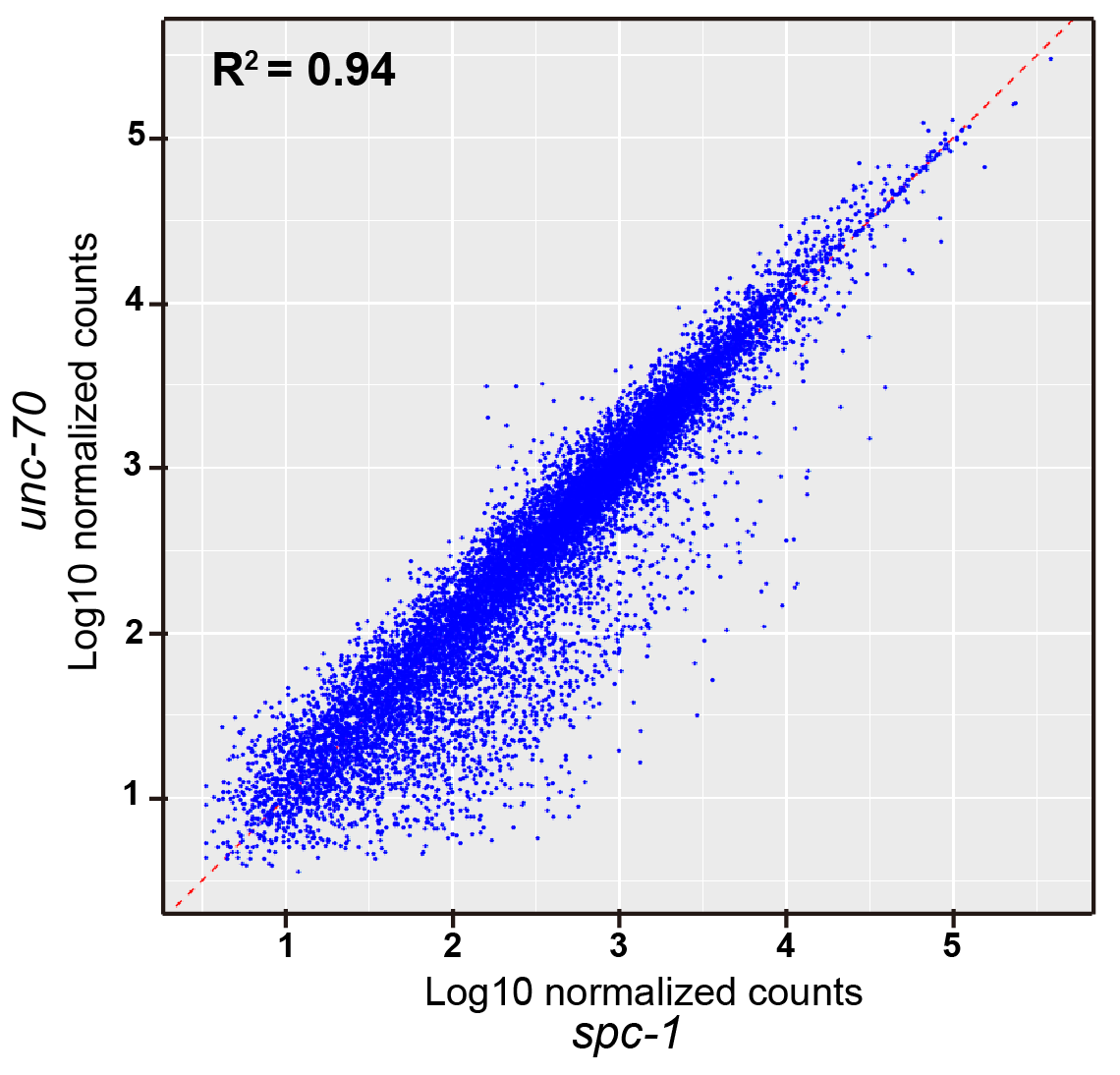

Supplement: S4 Fig — (TIF) [file pbio.3000369.s005.tif]

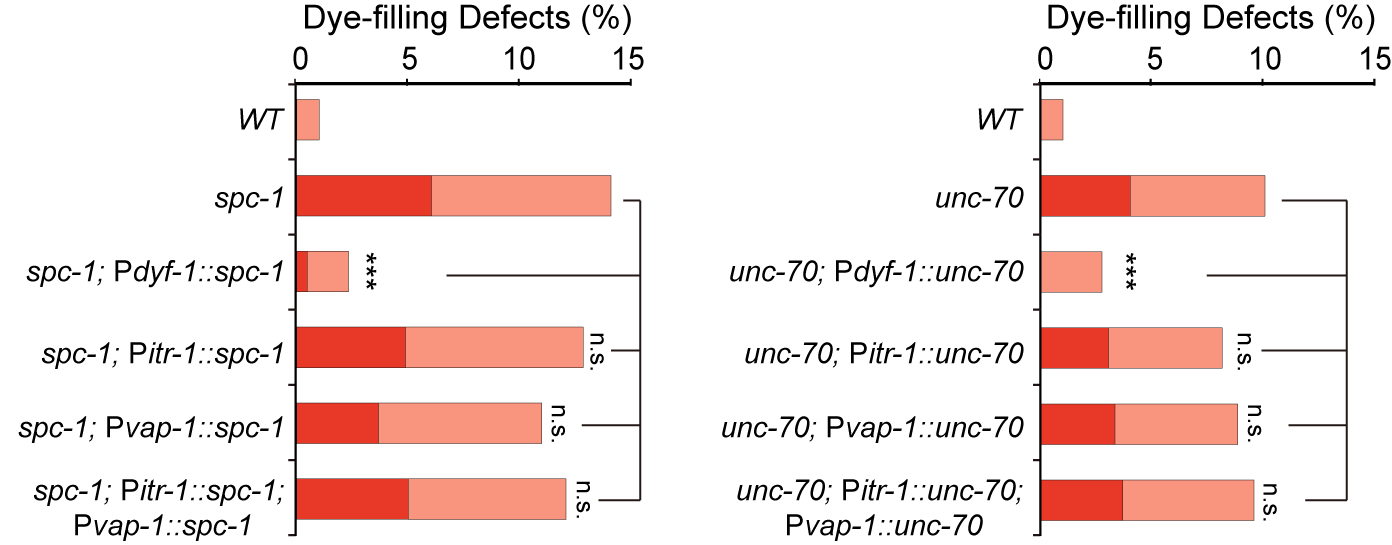

Supplement: S5 Fig — Quantifications of the animals showing no staining (red) or weak staining (light red) defects in the dye-filling assay from WT, mutant animals, or their rescue animals (N = 117–273). Statistical significances between underlined pairs are based on Student t test, ***p < 0.001. Data associated with this figure can be found in S1 Data. n.s., not significant; WT, wild type. (TIF) [file pbio.3000369.s006.tif]

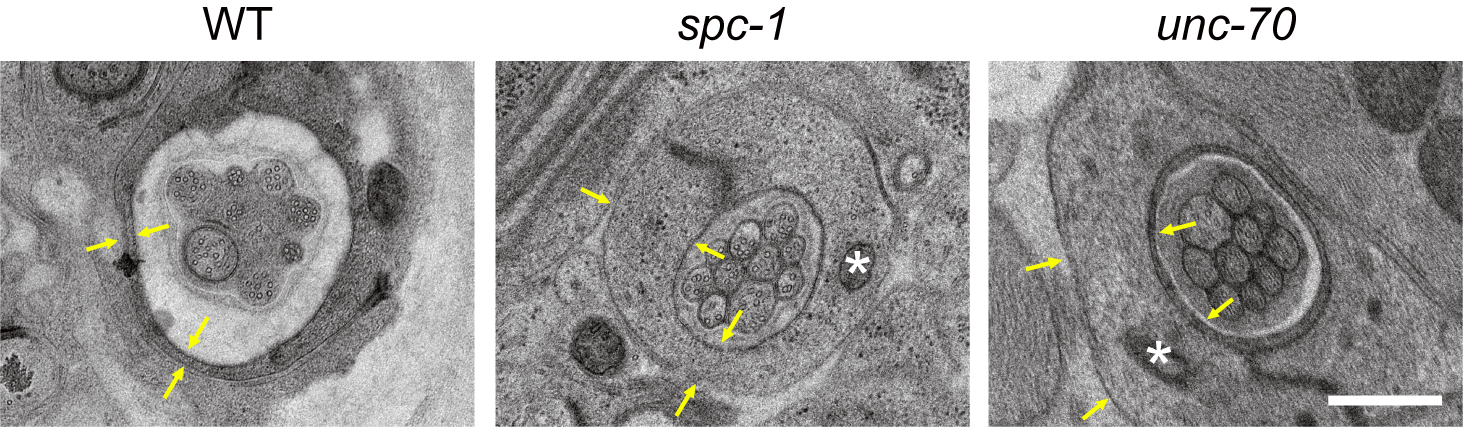

Supplement: S6 Fig — Yellow arrows indicate the boundary of socket cells. White asterisks indicate that vesicles accumulated in socket cells. TEM, transmission electron microscope. (TIF) [file pbio.3000369.s007.tif]

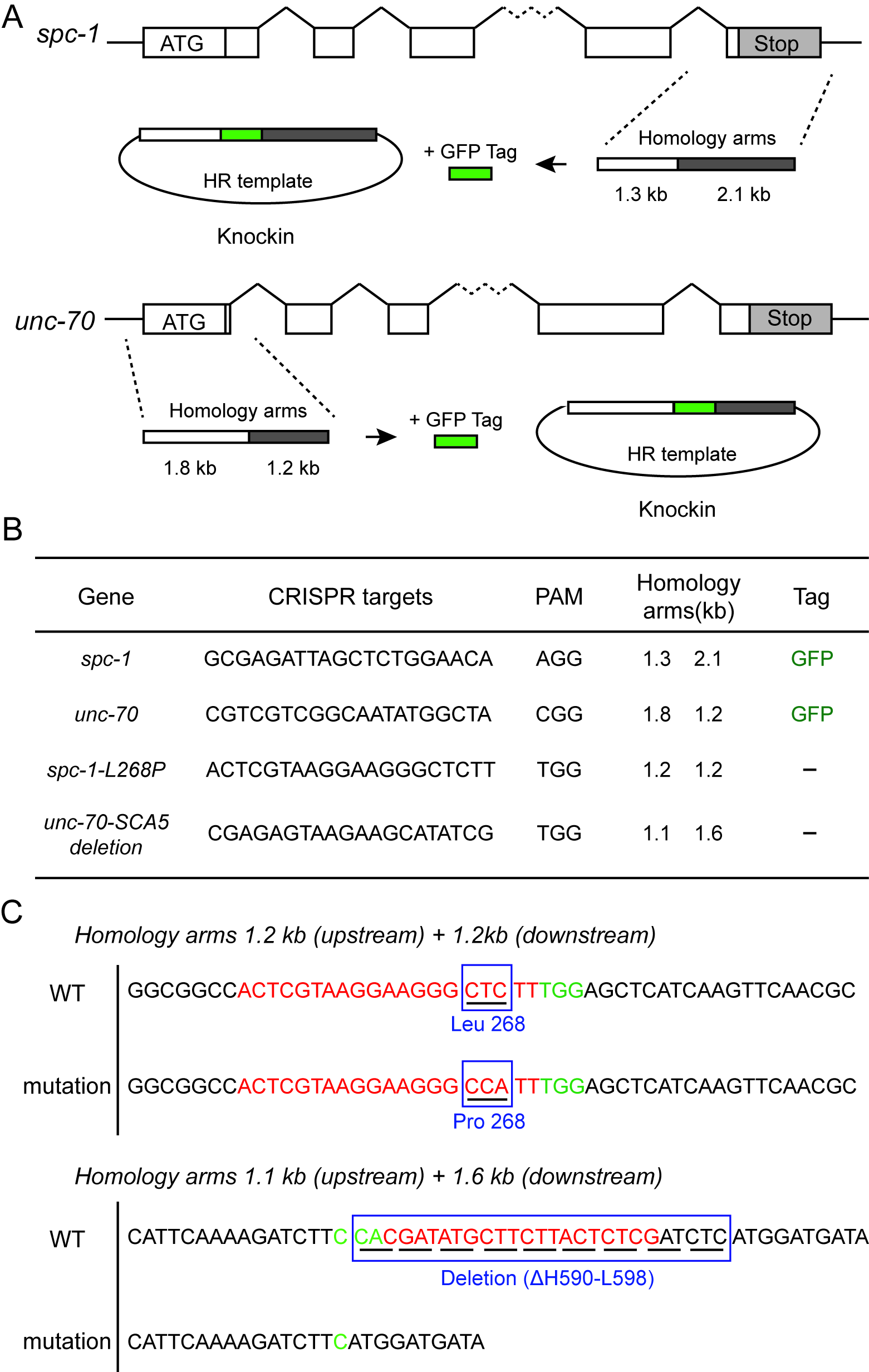

Supplement: S7 Fig — (A) Schematic design of the CRISPR-Cas9–assisted KI for C. elegans. sgRNAs targeting the 5ʹ or 3ʹ ends of genes and approximately 1 kb homology arms and fluorescent tags were inserted into HR templates. (B) Summary of CRISPR targets, homology arms, and tags. (C) Construction of HR templates to generate disease-related mutation KI strains. CRISPR targets and PAM motifs in WT genomes are colored in red and green, respectively. Disease-associated spc-1 (L268P) and unc-70 (ΔH590-L598) mutations in templates are colored in blue. CRISPR-Cas9, clustered regularly interspaced short palindromic repeats- Cas9; HR, homology recombination; KI, knock-in; PAM, protospacer adjacent motif; sgRNA, single-guide RNA; WT, wild type. (TIF) [file pbio.3000369.s008.tif]
