# Supplementary material for: Spectrin-based membrane skeleton supports ciliogenesis
Source: PLoS Biol. 2019 Jul 12;17(7):e3000369. doi: 10.1371/journal.pbio.3000369 (PMC6655744; doi:10.1371/journal.pbio.3000369)
Supplement: S1 Table — CRISPR, clustered regularly interspaced short palindromic repeats. (DOCX) [file pbio.3000369.s014.docx]

| **Table S1 Targets of CRISPR and primers for molecular analysis** | | |
| --- | --- | --- |
| **Gene** | **CRISPR-Cas9 targets (PAM)** | **Primers (For: forward; Rev: reverse)** |
| *spc-1 knock in* | sg1: GCGAGATTAGCTCTGGAACA **AGG** | For:  ATTAGCTCTGGAACAGTTTTAGAGCTAGAAATAGCAAG |
|  |  | Rev:  TGTTCCAGAGCTAATCTCGCCAAGACATCTCGCAATAGG |
|  | *sg2:ACAATTGGCGAGATTAGCTC* **TGG** | For:  TGGCGAGATTAGCTCGTTTTAGAGCTAGAAATAGCAAG |
|  |  | Rev:  GAGCTAATCTCGCCAATTGTCAAGACATCTCGCAATAGG |
| *unc-70 knock in* | *sg1:*CGTCGTCGGCAATATGGCTA **CGG** | For:  TAGCCATATTGCCGACGACGCAAGACATCTCGCAATAGG |
|  |  | Rev:  TCGGCAATATGGCTAGTTTTAGAGCTAGAAATAGCAAG |
|  | *sg2:* GCGAAACGTCGTCGGCAATA **TGG** | For:  TATTGCCGACGACGTTTCGCCAAGACATCTCGCAATAGG |
|  |  | Rev:  ACGTCGTCGGCAATAGTTTTAGAGCTAGAAATAGCAAG |
| *spc-1-L268P knock in* | *sg1:* ACTCGTAAGGAAGGGCTCTT **TGG** | For:  TAAGGAAGGGCTCTTGTTTTAGAGCTAGAAATAGCAAG |
|  |  | Rev:  AAGAGCCCTTCCTTACGAGTCAAGACATCTCGCAATAGG |
|  | *sg2:* AAAGAGCCCTTCCTTACGAG **TGG** | For:  CTCGTAAGGAAGGGCTCTTTcaagacatctcgcaatagg |
|  |  | Rev:  GCCCTTCCTTACGAGGTTTTAGAGCTAGAAATAGCAAG |
| *unc-70-ΔH590-L598 knock in* | *sg1:* CGAGAGTAAGAAGCATATCG **TGG** | For:  CGATATGCTTCTTACTCTCGCAAGACATCTCGCAATAGG |
|  |  | Rev:  GTAAGAAGCATATCGGTTTTAGAGCTAGAAATAGCAAG |
|  | *sg2:* GCTTCTTACTCTCGATCTCA **TGG** | For:  TGAGATCGAGAGTAAGAAGCCAAGACATCTCGCAATAG |
|  |  | Rev:  TTACTCTCGATCTCAGTTTTAGAGCTAGAAATAGCAAG |
