# Supplementary material for: Spectrin-based membrane skeleton supports ciliogenesis
Source: PLoS Biol. 2019 Jul 12;17(7):e3000369. doi: 10.1371/journal.pbio.3000369 (PMC6655744; doi:10.1371/journal.pbio.3000369)
Supplement: S2 Table — qPCR, quantitative polymerase chain reaction. (DOCX) [file pbio.3000369.s015.docx]

| **Table S2 Oligonucleotides for qPCR** | | |
| --- | --- | --- |
| **Gene** | **Primers (For: forward)** | **Primers (Rev: reverse)** |
| *act-1* | For: CCGTCTTGACTTGGCTGGA | Rev: CTCAGCGGTGGTGGTGAAA |
| *ift-81* | For: AACGGCAGAAGGTCCTGAACAT | Rev: GTTCGTCGCAACTTCGTCCTC |
| *dyf-11* | For: GCCACAAGTGGTAGTCGAACT | Rev: CTAGCGGTTCTATCACCATCTTCC |
| *osm-6* | For: ATGCCTCCATTTTCAGACGA | Rev: TTGCCACACCACGAAAACC |
| *che-13* | For: GCGACTGAATCGAAGAATCCACTC | Rev: GAAGACGCCAATCTTTTGCATCC |
| *ifta-1* | For: CTCTATGATCGCTCTCGCAGCA | Rev: GGGTTCACTGGAGGATTTTCTGAG |
| *ifta-2* | For: GGCCAACAAAAGGAGTCAGAATAC | Rev: ATTGCTGGCCAGCAGTCTTCG |
| *che-3* | For: GGAACATTGCAGCTGTTCGAATC | Rev: TCCTTGAAGCAAGAGCCCTTG |
| *bbs-2* | For: TGCTCAGTTGAGATGGAACAACCA | Rev: AGACAGACGCTCCGTACTGCA |
| *bbs-5* | For: CAGACTACTTCGCTAGTCACAAGA | Rev: GGCTCTGCTGATTGATGAGATCG |
| *odr-10* | For: AGCGGCTGTCACCAATGTACTAAA | Rev: CACGTGGCATATCCGCAGTAG |
| *mks-6* | For: GAGGAAAGACGAACGAGCTCGA | Rev: CCTACCCACTGCCACATCAGTA |
