# Supplementary material for: Spectrin-based membrane skeleton supports ciliogenesis
Source: PLoS Biol. 2019 Jul 12;17(7):e3000369. doi: 10.1371/journal.pbio.3000369 (PMC6655744; doi:10.1371/journal.pbio.3000369)
Supplement: S3 Table — (DOCX) [file pbio.3000369.s016.docx]

| **Table S3 Primers and plasmids used for plasmid cloning in this study** | | | |
| --- | --- | --- | --- |
| **Plasmid Name** | **Primer 5’** | **Primer 3’** | **Notes** |
| pDD162-P*eft-3::*Cas9 + P*U6:: spc-1 knock in sg1* | TGTTCCAGAGCTAATCTCGCCAAGACATCTCGCAATAG | ATTAGCTCTGGAACAGTTTTAGAGCTAGAAATAGCAA | PCR from pDD162-P*eft-3*::Cas9+P*U6::Empty sgRNA* |
| pDD162-P*eft-3*::Cas9 + P*U6::spc-1 knock in sg2* | GAGCTAATCTCGCCAATTGTCAAGACATCTCGCAATAG | TGGCGAGATTAGCTCGTTTTAGAGCTAGAAATAGCAA | PCR from pDD162-P*eft-3*::Cas9+P*U6::Empty sgRNA* |
| pDD162-P*eft-3*::Cas9 + P*U6:: unc-70 knock in sg1* | TAGCCATATTGCCGACGACGCAAGACATCTCGCAATA | TCGGCAATATGGCTAGTTTTAGAGCTAGAAATAGCAA | PCR from pDD162-P*eft-3*::Cas9+P*U6::Empty sgRNA* |
| pDD162-P*eft-3*::Cas9 + P*U6:: unc-70 knock in sg2* | TATTGCCGACGACGTTTCGCCAAGACATCTCGCAATAG | ACGTCGTCGGCAATAGTTTTAGAGCTAGAAATAGCAA | PCR from pDD162-P*eft-3*::Cas9+P*U6::Empty sgRNA* |
| pDD162-P*eft-3*::Cas9 + P*U6:: spc-1-L268 knock in sg1* | AAGAGCCCTTCCTTACGAGTCAAGACATCTCGCAATAG | TAAGGAAGGGCTCTTGTTTTAGAGCTAGAAATAGCAA | PCR from pDD162-P*eft-3*::Cas9+P*U6::Empty sgRNA* |
| pDD162-P*eft-3*::Cas9 + P*U6:: spc-1-L268 knock in sg2* | TTCCTTACGAGTGGCCGCCACAAGACATCTCGCAATA | GCCACTCGTAAGGAAGTTTTAGAGCTAGAAATAGCAA | PCR from pDD162-P*eft-3*::Cas9+P*U6::Empty sgRNA* |
| pDD162-P*eft-3*::Cas9 + P*U6:: unc-70-ΔH590-L598 knock in sg1* | CGATATGCTTCTTACTCTCGCAAGACATCTCGCAATA | GTAAGAAGCATATCGGTTTTAGAGCTAGAAATAGCAA | PCR from pDD162-P*eft-3*::Cas9+P*U6::Empty sgRNA* |
| pDD162-P*eft-3*::Cas9 + P*U6:: unc-70-ΔH590-L598 knock in sg2* | TGAGATCGAGAGTAAGAAGCCAAGACATCTCGCAATA | TTACTCTCGATCTCAGTTTTAGAGCTAGAAATAGCAAG | PCR from pDD162-P*eft-3*::Cas9+P*U6::Empty sgRNA* |
| pPD95.77*-spc-1-5’ arm::gfp knock in* | GAAGAGTAATTGGACCACAATGCGAGTGGCGTTTC | GTACCGGTAGAAAAA GGAAGAGATCCGTGCCTT | The 5' arm sequences were amplified from N2 and cloned into pPD95.77 via In-Fusion Advantage PCR Cloning Kit. |
| pPD95.77*-spc-1-5’ arm::gfp-3’ arm knock in* | AGACCCAAGCTTGGTACCATGAGT | CTATTTGTATAGTTCATCCATGCC | The 3' arm sequences were amplified from N2 and cloned into pPD95.77-*spc-1-5’ arm::gfp* knock in via In-Fusion Advantage PCR Cloning Kit. |
| pPD*95.77-unc-70-5’ arm::gfp knock in* | GAAGAGTAATTGGACCAGACGTTCACCGGAAAGAACTGCG | GTACCGGTAGAAAAACCACCCATCACTCTCTCGTAACCTC | The 5' arm sequences were amplified from N2 and cloned into pPD95.77 via In-Fusion Advantage PCR Cloning Kit. |
| pPD95.77-*unc-70-5’ arm::gfp-3’ arm knock in* | GGAAGTGGTAGCGGTATGGCTACGGTGAGTTTTTT | ATGAGTAAAGGAGAAGAACTTTTC | The 3' arm sequences were amplified from N2 and cloned into pPD95.77-*unc-70-1-5’ arm::gfp knock in* via In-Fusion Advantage PCR Cloning Kit. |
| pPD95.77*-spc-1-5’ arm::7×gfp11 knock in* | GAAGAGTAATTGGACCACAATGCGAGTGGCGTTTC | GTACCGGTAGAAAAA GGAAGAGATCCGTGCCTTGC | The 5' arm sequences were amplified from N2 and cloned into pPD95.77 via In-Fusion Advantage PCR Cloning Kit. |
| pPD95.77*-spc-1-5’ arm::7×gfp11-3’ arm knock in* | GGTGATACCGGCAGCATTGACATATTCG | ATGCGTGACCACATGGTCCTTCATGA | The 3' arm sequences were amplified from N2 and cloned into pPD95.77-*spc-1-5’ arm:: 7×gfp11* knock in via In-Fusion Advantage PCR Cloning Kit. |
| pPD95.77-*spc-1-L268P arm knock in* | GAACGAGTCAGCACGAGCATCAAT | GCCCTCTGGGATAAGCTCTTCTTCAAACTG | The *spc-1* sequences were amplified from N2 and cloned into pPD95.77 via In-Fusion Advantage PCR Cloning Kit. |
| pPD95.77*-spc-1-L268P knock in repair template* | CCAAATGGCCCTTCCTTACGAGTGGCCGCCAAAGT | GGAAGGGCCATTTGGAGCTCATCAAGTTCAACGCT | PCR on pPD95.77*-spc-1-L268P* knock in repair template |
| pPD95.77*-unc-70-ΔH590-L598 arm knock in* | GCATGGCAATCCCTTGAGAAGGCAGAACACGAAC | GAAGAGTAATTGGACTGGCTCTTCCTCTGAGGCAAC | The *unc-70* sequences were amplified from N2 and cloned into pPD95.77 via In-Fusion Advantage PCR Cloning Kit. |
| pPD95.77-*unc-70 ΔH590-L598 knock in repair template* | ATGAGATCCATTGACAACTCCAATCGAACTCTGCG | GTCAATGGATCTCATGGATGATATTAAGAGCAGAC | PCR on pPD95.77*-unc-70-ΔH590-L598* arm knock in |
