# Supplementary material for: Spectrin-based membrane skeleton supports ciliogenesis
Source: PLoS Biol. 2019 Jul 12;17(7):e3000369. doi: 10.1371/journal.pbio.3000369 (PMC6655744; doi:10.1371/journal.pbio.3000369)
Supplement: S4 Table — SOE, splicing by overlapping extension. (DOCX) [file pbio.3000369.s017.docx]

| **Table S4 Primers for SOEing PCR** | |
| --- | --- |
| **PCR Products** | **Primers (Forward/ Reverse)** |
| P*dyf-1* | For: AAGGATTCCCGTAAAATCATGAGA  Rev: TGCTATATTTCTGTGTAAGCTTGTCAAA |
| P*itr-1* | For: CGTTCCCGAGCATTATGAATGTAAG  Rev: GATTTTGATGATTTTGAGGAACAAATT |
| P*vap-1* | For: CCTACTGTAGAGGAGATGTTGAGCAATA  Rev: GCGTGCGTTCATTTTCACAG |
| *spc-1* | For: CTGTGTAAGCTTGTCAAAATGGCTGATTCGAATGACAC  Rev: ACAGAGAACATTCTGGTAACACG |
| *unc-70* | For: CTGTGTAAGCTTGTCAAAATGGCTACGGTGAGTTTTTT  Rev: TCACCTTTGGTTCACTTTGG |
| P*dyf-1::spc-1* | For: AAACACGAAAAACCTCTACAAACAT  Rev: TATAGCTGCCAAGTGCGATG |
| P*dyf-1::unc-70* | For: AAACACGAAAAACCTCTACAAACAT  Rev: TTCTGTGGCAGCTTTATTTC |
| P*itr-1::spc-1* | For: CACTGAAAAATGTACAGGAAACTC  Rev: TATAGCTGCCAAGTGCGATG |
| P*itr-1::unc-70* | For: CACTGAAAAATGTACAGGAAACTC  Rev: TTCTGTGGCAGCTTTATTTC |
| P*vap-1::spc-1* | For: CACTTTGCCATAAACTCCTGC  Rev: TATAGCTGCCAAGTGCGATG |
| P*vap-1::unc-70* | For: CACTTTGCCATAAACTCCTGC  Rev: TTCTGTGGCAGCTTTATTTC |
