# Supplementary material for: Spectrin-based membrane skeleton supports ciliogenesis
Source: PLoS Biol. 2019 Jul 12;17(7):e3000369. doi: 10.1371/journal.pbio.3000369 (PMC6655744; doi:10.1371/journal.pbio.3000369)
Supplement: S5 Table — (DOCX) [file pbio.3000369.s018.docx]

| **Table S5. *C. elegans* strains used in this study** | | | |
| --- | --- | --- | --- |
| **Strain name** | **Genotype** | **Method** | **Resource** |
| N2 | Wild type | - | CGC |
| GOU2936 | *cas815[spc-1::gfp knock in]* | Microinjection | This study |
| GOU3238 | *cas971[spc-1-L260P knock in]* | Microinjection | This study |
| GOU3599 | *cas1049[spc-1-L268P::gfp]* | Microinjection | This study |
| GOU3519 | *cas1047[spc-1::7×gfp11 knock in]* | Microinjection | This study |
| GOU3544 | *cas1047[spc-1::7×gfp11 knock in]; casEx5000[*P*dyf-1:: gfp1-10,pRF4(+)]* | Genetic cross | This study |
| GOU3103 | *cas962[gfp::unc-70 knock in]* | Microinjection | This study |
| GOU3237 | *cas983[unc-70-ΔH590-L598 knock in]* | Microinjection | This study |
| GOU3601 | *cas1050[unc-70-ΔH590-L598::gfp]* | Microinjection | This study |
| GOU3667 | *cas971[spc-1-L260P knock in]; cas1013[che-2::3xgfp]* | Genetic cross | This study |
| GOU3668 | *cas983[unc-70-ΔH590-L598 knock in]; cas1013[che-2::3xgfp]* | Genetic cross | This study |
| GOU3664 | *cas971[spc-1-L260P knock in]; mnIs17[osm-6::gfp]* | Genetic cross | This study |
| GOU3665 | *cas983[unc-70-ΔH590-L598 knock in]; mnIs17[osm-6::gfp]* | Genetic cross | This study |
| GOU3605 | *cas1070[*P*dyf-1-myri-scarlet; pRF4(+)]; cas971[spc-1-L260P knock in]* | Genetic cross | This study |
| GOU3608 | *cas1070[*P*dyf-1-myri-scarlet; pRF4(+)]; cas983[unc-70-ΔH590-L598 knock in]* | Genetic cross | This study |
| GOU3642 | *cas1047[spc-1::7×gfp11 knock in];* *casEx5000[*P*dyf-1:: gfp1-10,pRF4(+)]; casIs550[*P*dyf-1::osm-6::mCherry; unc-76(+)]* | Genetic cross | This study |
| GOU3677 | *casEX5756[*P*dyf-1::spc-1;* P*egl-17::mCherry-Myri;* P*egl-17::mCherry::TEV-S::his-24];cas971[spc-1-L260P knock in];* | Microinjection | This study |
| GOU3678 | *casEX5757[*P*dyf-1::unc-70;* P*egl-17::mCherry-Myri;* P*egl-17::mCherry::TEV-S::his-24];cas983[unc-70-ΔH590-L598 knock in]* | Microinjection | This study |
| GOU3679 | *casEX5758[*P*itr-1:: spc-1;* P*vap-1:: spc-1;* P*egl-17::mCherry-Myri;* P*egl-17::mCherry::TEV-S::his-24];cas971[spc-1-L260P knock in];* | Microinjection | This study |
| GOU3680 | *casEX5759[*P*itr-1::unc-70;* P*vap-1::unc-70;* P*egl-17::mCherry-Myri;* P*egl-17::mCherry::TEV-S::his-24];*  *cas983[unc-70-ΔH590-L598 knock in]* | Microinjection | This study |
| GOU3681 | *casEX5760[*P*itr-1::spc-1;* P*egl-17::mCherry-Myri;* P*egl-17::mCherry::TEV-S::his-24]; cas971[spc-1-L260P knock in]* | Microinjection | This study |
| GOU3682 | *casEX5761[*P*vap-1::spc-1;* P*egl-17::mCherry-Myri;* P*egl-17::mCherry::TEV-S::his-24]; cas971[spc-1-L260P knock in]* | Microinjection | This study |
| GOU3683 | *casEX5762[*P*itr-1::unc-70;* P*egl-17::mCherry-Myri;* P*egl-17::mCherry::TEV-S::his-24]; cas983[unc-70-ΔH590-L598 knock in]* | Microinjection | This study |
| GOU3684 | *casEX5763[*P*vap-1::unc-70;* P*egl-17::mCherry-Myri;* P*egl-17::mCherry::TEV-S::his-24]; cas983[unc-70-ΔH590-L598 knock in]* | Microinjection | This study |
